# Efficacy of *Aster chinensis* aerial parts metabolites in BALB/c mice model of Imiquimod-induced psoriasis skin inflammation

**DOI:** 10.1007/s10787-025-01652-x

**Published:** 2025-03-12

**Authors:** Mai S. Hendawy, Mona M. Hashem, Ahmed A. Zaki, Mostafa A. Rabie, Nesrine S. El Sayed, Riham Salah El Dine, Ali M. El-Halawany

**Affiliations:** 1 Department of Pharmacognosy, Faculty of Pharmacy, Horus University, New Damietta, 34518 Egypt; 2 Department of Pharmacognosy, Faculty of Pharmacy, Cairo University, Kasr El-Aini Street, Cairo, 11562 Egypt; 3 Department of Pharmacognosy, Faculty of Pharmacy, Mansoura University, Mansoura, 35516 Egypt; 4 Department of Pharmacology & Toxicology, Faculty of Pharmacy, Cairo University, Kasr El-Aini Street, Cairo, 11562 Egypt

**Keywords:** *A. chinensis*, *A. Squamatus*, UHPLC-MS/MS, Imiquimod-induced psoriasis, Inflammatory mediators, Histopathological examination

## Abstract

Using a bioassay-guided fractionation approach, the most potent anti-psoriatic components of *Aster squamatus* herb, *Aster chinensis* stalks, and *Aster chinensis* flowers, cultivated in Egypt, were identified and evaluated against Imiquimod (IMQ)-induced psoriasis in female BALB/c mice and compared to standard drug, mometasone. The topical application of *A. chinensis* stalk methanolic extract exhibited the strongest anti-psoriatic effects against IMQ-induced psoriasis model, as evidenced by improvements in psoriasis area severity index (PASI) score, histopathological analysis, and spleen index. Further fractionation of *A. chinensis* stalk methanolic extract using petroleum ether, methylene chloride, ethyl acetate, and *n*-butanol revealed that the methylene chloride fraction (MCF) was the most potent. Indeed, MCF significantly reduced the PASI score, alleviated histopathological changes, and restored spleen index. Mechanistically, MCF exerted its anti-psoriatic effects by suppressing inflammation, evidenced by decreased TLR-4 gene expression and lower levels of HMGB1 and NFκBp65 protein contents. Additionally, MCF reduced serum levels of pro-inflammatory cytokines interleukin (IL)-1β, IL-6, IL-23, and IL-17 while mitigating oxidative stress through increased superoxide dismutase (SOD) activity and reduced malondialdehyde (MDA) content. Notably, the efficacy of MCF was comparable to that of mometasone, with no significant differences observed. In parallel, the chemical profile of the MCF was analyzed using UHPLC-MS/MS techniques in negative and positive ionization full scan modes. MCF of *A. chinensis stalk* could be used a potential therapeutic agent for psoriasis.

**Supplementary Information:**

The online version contains supplementary material available at 10.1007/s10787-025-01652-x.

## Introduction

Psoriasis is a chronic autoimmune inflammatory disease that primarily affects skin, nails, and sometimes joints. It is characterized by chronic skin inflammation, excessive keratinocyte proliferation, and itchy, silvery scaly and erythematous lesions on the skin (Griffiths and Barker 2007; Rachakonda et al. 2014). Psoriasis is characterized by a key clinical feature, the hyperproliferation of keratinocytes, driven by T-cell activation. In normal skin, the proliferation rate of keratinocytes is balanced by differentiation and shedding. However, in psoriatic lesions, this balance is disrupted, leading to a proliferation rate approaching 100%, compared to approximately 60% in healthy skin (Gudjonsson et al. 2004). The pathogenesis of psoriasis involves a complex interplay between cytokines and immune cells. The release of interleukin (IL)-23 and, to a lesser extent, IL-12, promotes the differentiation of T helper cells (Th) into pro-inflammatory subsets, particularly Th17 and Th22. These Th cells, in turn, produce a cascade of cytokines, including IL-17, interferon-gamma (IFN-γ), tumor necrosis factor-alpha (TNF-α), and IL-22, which directly stimulate keratinocyte hyper proliferation and contribute to the inflammatory state characteristic of psoriasis (Lowes et al. 2014). Van der Fits et al. (2009) firstly prescribed Imiquimod-induced psoriasis as a fast, effective, and appropriate psoriasis model (van der Fits et al. 2009). Imiquimod (IMQ), an imidazoquinoline heterocyclic amine, activate the immune system via the production of IFN-α, TNF-α, IL-6, IL-8, IL-17, and IL-23. It is also acting as an agonist for Toll-like receptor (TLR)-7 and 8. Due to these properties, it finds applications as an antiviral for anogenital warts caused by the human papilloma virus, and an antitumor agent for treatment basal cell carcinoma, and actinic keratosis (Hanna et al. 2016; van der Fits et al. 2009). IMQ-induced inflammatory lesions in mice look like human psoriasis lesions (van der Fits et al. 2009).

Nowadays, medicinal plants play an important role in health care, food, cosmetics, nutraceuticals, and drug discovery (Devkota and Aftab 2022). Family Asteraceae (Compositae), also named as sunflower family, is one of the biggest families of flowering plants consisting of about 1600 genera and 32,000 species (Boulechfar et al. 2014; Li et al. 2022).

Studies on the phytochemical profiles of *Aster* species suggest the presence of various classes of bioactive secondary metabolites, such as flavonoids, terpenoids, phenolics, glycosides, fatty acids, lignans, and sterols (Devkota and Aftab 2022; Li et al. 2022).

*A. squamatus* (Spreng.) Hieron is an annual herb native to South America (Kalam et al. 2020). It is characterized by multiple stems with soft, woody leaves and white flowers that bloom from September to November (Boulechfar et al. 2014). The plant has become widely naturalized in tropical and subtropical regions around the world, except for Antarctica. Interestingly, *A. squamatus* can also be found growing wild along the banks of the Nile River in Egypt. *A. squamatus* has a history of use in traditional medicine for its potential antidiarrhoeic, antineoplastic, and cicatrizing effects (Sperotto et al. 2002). Phytochemical studies of *A.squamatus* reported the presence of steroids, flavonoids as squamatin, ternatin, ramnetim, kaempferol, baicalein, and quercetin, terpenes, phenols as cinnamic, caffeic and sinapic acids, amino groups, saponins, and pyrogallol and catechol tannins (Sperotto et al. 2002; Ghedini et al. 2002).

*A. chinensis* L. belongs to family Asteracea, also known as *Callistephus chinensis* (L.) Nees, native to China, this annual plant is widely cultivated worldwide as a winter ornamental plant in gardens, floral decoration, bouquets, and as a cut flower (Bhargav et al. 2018). The name "Callistephus" comes from two Greek words, kalistos, which means ‘most beautiful’, and stephos, which means ‘a crown’, that referring to the flower head *A. chinensis* thrives in cool weather with full sun or partial shade. It features ovate leaves with a dentate margin, and produces large (7–12 cm) flowers, with a single row of petals or a fully double flower, that bloom in March, in a variety of colors, including White, pink, red, blue, purple, and yellow (Bhargav et al. 2018; Biswas et al. 2021). Interestingly, traditional Chinese medicine utilized *A. chinensis* for pain relief and flu prevention through consumption as a tea (Bi et al. 2014). The investigation of chemistry of *A. chinensis* showed several classes such as flavonoids (apigenin, catechin, epicatechin, hesperidin, myricetin, luteolin, and naringenin) (Bhargav et al. 2018), isoaurones as (*Z*)-4′,4,10-trihydroxy-siamaurone and (*E*)-4′,4,10-trihydroxy-siamaurone (Zhang et al. 2016), sesquiterpene such as callistephus A, and callistephus B (Zhang et al. 2015), and coumarin as umbelliferone (Bhargav et al. 2018).

A comprehensive review of the literature revealed no prior investigations into the anti-psoriatic potential of stalks from *A. squamatus* and *A. chinensis*, nor its flowers. This study, therefore, is the first to explore their possible application in the treatment of psoriasis. Following the initial assessment of anti-psoriatic activity, bio-guided fractionation of *A. chinensis* stalks alcoholic extract was performed. A further study was performed on the most active fraction from *A. chinensis* stalks to focus on elucidating the bioactive components that may be responsible for the observed effects via the UHPLC-MS/MS technique.

## Materials and methods

### Collection of plant materials

The whole parts of *A. squamatus* herb were collected in September 2021 from Mansoura, Egypt. It is growing wild on the river Nile banks. On other hand, *A. chinensis* stalks and flowers were collected in March 2022 from private botanical garden at Mansoura, Egypt. Botanical identification was kindly identified by Prof. Dr. Ibrahim Mashaly, Prof. of Ecology, Faculty of Science, Mansoura University, and Ass. Prof. Dr. Mahmoud Makram Kasem, Ass. Prof. of Ornamental, Aromatic and Medicinal plants, Faculty of Agriculture, Mansoura University, Egypt. Voucher specimens were deposited at the herbarium of the Department of Pharmacognosy, Faculty of Pharmacy, Cairo University, Cairo, Egypt (Code (14-6-23-F), and (13-6-23-F)) for *A. squamatus* herb, and *A. chinensis* stalks and flowers, respectively.

### Chemicals and reagents

#### Chemicals used for chromatography

Organic solvents used for extraction were petroleum ether, methylene chloride, ethyl acetate, *n-*butanol, and MeOH purchased from El-Nasr Company for Pharmaceutical Chemicals, Egypt. According to UHPLC-MS/MS, mobile phase A (0.1% formic acid in water) and B (acetonitrile) were used, all solvents used were of UHPLC-MS/MS grade.

### Extraction procedure and fractionation

Firstly, the flowers of *A.chinensis* were separated from their stalks. *A.squamatus*, *A.chinensis* stalks, and *A.chinensis* flowers were air-dried at room temperature and then powdered. The powdered plant materials were (600, 490, 301 g respectively), exhaustively extracted by maceration in methanol at room temperature. Briefly, 50 g of dry powdered plant materials were extracted with 600 mL of methanol. The collected methanolic extracts were evaporated under reduced pressure to give semisolid residue. The dried methanolic extracts of *A.squamatus*, *A.chinensis* stalks, and *A.chinensis* flowers were 290, 220.36, 145.76 g, respectively. The dried methanolic extracts of *A.chinensis* stalks, demonstrating the strongest anti-psoriatic activity, re-dissolved in a minimum volume of methanol, and diluted with a minimum volume of distilled water, then it was subjected to sequential fractionation using solvents of increasing polarity: (1.5 L) petroleum ether, (500 mL) methylene chloride, (1 L) ethyl acetate, and (5 L) *n*-butanol. Each solvent was evaporated to dryness under reduced pressure giving a dried residue as following: (12 g) petroleum ether, (3 g) methylene chloride, (7 g) ethyl acetate, and (50 g) *n*- butanol. For UHPLC-MS/MS, a 1 mg of the methylene chloride fraction of *A.chinensis* stalks was suspended in 1 mL of methanol.

### UHPLC-MS/MS analysis of sample secondary metabolites

3 µl of methylene chloride fraction of *A.chinensis* stalks dissolved in 100% methanol prepared was subjected to chromatographic separation using an I-Class UPLC system (Waters Corporation, Milford, USA). Chromatographic separation was carried out at 40 °C, using a Waters HSS T3 column (1.0 mm × 100 mm, 1.8 μm) with mobile phases A (0.1% formic acid in water) and B (acetonitrile). The flow rate was set at 0.15 mL/min. The gradient profile was as follows: 0–1 min, 5%–5% B; 1–11 min, 5%–100% B; 11–19 min, 100% B; 19–20 min, 100%–5% B; 20–25 min, 5% B.

Mass spectrometric detection was carried out on Waters Synapt XS mass spectrometer (Waters Corporation, Milford, USA) equipped with an ESI source. The full scan data were acquired from 50 to 1200 Da, using a capillary voltage of 4.0 kV for positive ion mode and 3.0 kV for negative ion mode, sampling cone voltage of 30 V for positive ion mode and 35 V for negative ion mode, extraction cone voltage of 4.0 V, source temperature of 140 °C, cone gas flow of 50 L/h, desolvation gas (N^2^) flow of 1000 L/h and desolvation gas temperature of 450 °C. The collision voltage was set as 5.0 eV for low-energy scan and 25–50 eV for high-energy scan. Data were centroid and mass was corrected during acquisition using an external reference (Lock-Spray™) consisting of a 200 mg/mL solution of leucine enkephalin infused at a flow rate of 10 μL/min via a lockspray interface, generating a real-time reference ion of [M + H]^+^ (*m*/*z* 556.2771) in positive ion mode and [M − H]^−^ (*m*/*z* 554.2615) in negative ion mode to ensure accurate MS analysis. All data collected in centroid mode were obtained and used to calculate the accurate mass and composition of relative target ions with Mass Lynx™ V4.2 software (Waters).

### Animals

Eighty-four female BALB/c mice, weighing between 17 and 23 g, were sourced from the National Organization Center in Giza, Egypt. The mice underwent a 1-week acclimatization period at the animal house of the Faculty of Pharmacy, Cairo University, where temperature (23 ± 2 °C), humidity (65–70%), and a 12/12-h light/dark cycle was meticulously controlled. Prior to the experiment, the mice were shaved using a sterilized electronic razor, and hair removal cream was applied to their backs 2 days in advance to ensure complete hair removal.

### Ethical statement

The research followed the guidelines outlined in the Guide for the Care and Use of Laboratory Animals by the US National Institutes of Health (NIH Publication No. 85-23, revised 2011) and received approval from the Research Ethics Committee of the Faculty of Pharmacy, Cairo University, Cairo, Egypt (Permit number MP(3255). Every effort was made to minimize animal suffering throughout the experiment.

### Experimental design

The experiment was conducted in three stages. *Initially*, the biological activity of three herbal extracts, *A. chinensis flower, stalk* and *A. Squamatus*, was compared. The mice were divided randomly into six groups (*n* = 5/group). These groups consisted of a *normal control* group receiving 60 mg/day of Vaseline (group 1), *a diseased group* treated with 62.5 mg/day of Imiquimod (IMQ) cream (Aldara® cream 5%; group 2) (Elgewelly et al. 2022), and a standard *group* treated with 62.5 mg/day of 5% IMQ cream and 60 mg/day of mometasone cream 0.1% (group 3) (Kamal et al. 2024). The remaining three groups (group 4–6) received the same dose of IMQ cream as group 2 but were treated daily with *A. chinensis flower extract* (Treat.1; 100 mg/kg; p.o.; group 4), *A. chinensis stalk extract* (Treat.2; 100 mg/kg; p.o.; group 5), and *A. Squamatus extract* (Treat.3; 100 mg/kg; p.o.; group 6). The doses were selected based on a previous study involving another species of the Aster family, which was used for wound healing in diabetic rats (Hyun et al. 2018). The treatment was carried out for 7 days, and the evaluation of the PASI score, spleen index, and histopathological examination revealed that *A. chinensis* stalk extract demonstrated superior efficacy compared to *A. chinensis* flower and *A. Squamatus* extracts in alleviating IMQ-Induced psoriasis-like symptoms.

*In the second stage*, various fractions of *A. chinensis* stalk were generated to identify which contributed to its biological activity. Each fraction was applied topically for 7 consecutive days using a gel formulation system. Specifically, 500 mg of each fraction was dissolved in 10 ml of a solvent mixture (EtOH/H2O/glycerin, 60/30/10 v/v), and these solutions were then gelled with 2.0% (w/v) HPC (Giannakou et al. 1998). Indeed, mice were randomly divided into six groups (*n* = 5/group): a normal control group receiving drug vehicle (group 1), a diseased group receiving 62.5 mg/day Imiquimod (IMQ) cream (group 2), and the remaining four groups (groups 3–6) received the same dose of IMQ cream as group 2, but were additionally treated daily with specific fractions: butanol fraction (50 mg/kg; group 3), methylene chloride fraction (50 mg/kg; group 4), petroleum ether fraction (50 mg/kg; group 5), and ethyl acetate fraction (50 mg/kg; group 6). Based on the evaluation of PASI scores and histopathological examination, the methylene chloride fraction of *A. chinensis* stalk exhibited the highest protective efficacy against IMQ-induced psoriasis-like pathological features.

*In the third stage*, the biochemical mechanisms behind the anti-psoriasis effects of the methylene chloride fraction of *A. chinensis* stalk were investigated. Mice were randomly divided into four groups (*n* = 6/group): a normal control group receiving drug vehicle (group 1), a diseased group receiving 62.5 mg/day Imiquimod (IMQ) cream (Aldara® cream 5%; group 2), a standard group was treated with 62.5 mg/day 5% IMQ cream and 60 mg/day mometasone cream 0.1% (group 3), and a treatment group receiving 62.5 mg/day 5% IMQ cream and the methylene chloride fraction *A. chinensis* stalk (group 4).

Four hours post-application of IMQ cream on the shaven back skin, measuring 2.5 cm × 2 cm, treatments were administered, and the experiment proceeded for 7 days. Throughout the study, all groups were monitored for signs of erythema, scaling, and skin thickness. On the eighth day, mice were anesthetized using a ketamine/xylazine mixture, and blood samples were collected from the retro-orbital sinus. Subsequently, the animals were euthanized, and spleen weight was measured to calculate the spleen index. The dorsal skin on the back (*n* = 3/group) was shaved and preserved in 10% formalin for histopathological examination. The remaining lesioned skin was promptly frozen in liquid nitrogen and stored at −80 °C for biochemical analysis.

### Severity scoring of skin inflammation (PASI score)

The severity of psoriatic lesions was evaluated using a modified version of the clinical psoriasis area and severity index (PASI), as described by Zhou et al. (2020). Erythema, scaling, and skin thickness were each assessed on a scale of 0 to 4, with scores representing the severity: 0 for none, 1 for mild, 2 for moderate, 3 for marked and 4 for very marked. These parameters were evaluated independently, and the cumulative score (ranging from 0 to 12) was used to indicate the severity of inflammation.

### Spleen index calculation

The body weights and the spleens of all mice were weighted, and the spleen index was determined using the following formula.

$$\text{Spleen index }=\frac{\text{spleen weight} (g)}{\text{Body weight} (g)}\times 10$$

#### Enzyme-linked immunosorbent assay (ELISA)

Blood samples collected from the retro-orbital sinus were spun in a centrifuge at 1000×*g* for 20 min to isolate clear sera. These sera were then analyzed using ELISA kits from MyBioSource (CA, USA) to quantify the levels of IL-1β (cat#: MBS701092), IL-6 (Cat: # MBS824703), IL-23 (cat#: MBS2023684), and IL-17 (cat#: MBS2020546). Additionally, skin tissues were homogenized with phosphate-buffered saline (PBS; pH 7.4) to create a 10% homogenate. This homogenate was used to measure the protein content of pS536 NFκB p65 (cat#: MBS9511033) and HMGB1 (Cat: # MBS2021855) using ELISA kits from MyBioSource. Concurrently, malondialdehyde (MDA; cat#: MD 2529) and superoxide dismutase (SOD; cat#: SD 2521) were determined through colorimetric assays using kits obtained from Bio diagnostic (Giza, EG). All procedures were performed following the manufacturers’ instructions, and the obtained values were normalized to protein content assessed via the (Bradford 1976).

#### Quantitative RT-PCR analysis

The tissue lysate was processed to extract total RNA using the RN easy Mini kit (Qiagen, Netherland). To ensure RNA quality, its purity was checked spectrophotometrically at OD 260/280 nm. The extracted RNA was then reverse transcribed into cDNA using an RT-PCR kit (Promega, Netherland). For qRT-PCR analysis, the SYBR Green Master Mix from Applied Bio systems (CA, USA) was employed following the recommended protocol. In each 25 μl reaction, 5 μl of cDNA was combined with 12.5 μl of SYBR Green mixture, 5.5 μl of RNase-free water, and 2 μl of specific primer (Table 1). PCR amplification involved 40 cycles of denaturation at 95 °C for 15 s, annealing at 60 °C for 60 s, and extension at 72 °C for 60 s. The relative expression of the target gene was normalized to β-actin using the 2^ (−ΔΔCT) (Livak and Schmittgen 2001).

**Table 1:** Primer sequence for TLR4 receptor

mRNA	Gene ID	Primer sequence 5′–3′
TLR4	21,898	Forward: AGCTTCTCCAATTTTTCAGAACTTC Reverse: TGAGAGGTGGTGTAAGCCATGC
β-Actin	11,461	Forward: CATTGCTGACAGGATGCAGAAGG Reverse: TGCTGGAAGGTGGACAGTGAGG

#### Histopathological examination

Skin tissue samples were preserved in 10% neutral buffered formalin for 72 h before being processed through various ethanol concentrations, followed by xylene for clearing, and infiltration with synthetic paraplast tissue embedding medium. After embedding, the samples were sectioned into 5-micron slices using a rotatory microtome and stained with hematoxylin and eosin, a standard staining method for microscopic examination of different skin layers. An experienced histologist, blinded to the experimental conditions, evaluated the tissue samples using a scoring system based on Abdelkader et al. (Abdelkader et al. 2021). Microscopic analysis was conducted using a Full HD microscopic imaging system with the Leica application module for histological analysis (Table 2).

**Table 2:** Scoring system of lesions detected by microscopically examination of tissue sections

−	Nil (no lesions were demonstrated)
+	Mild lesion recorded in less than 15% of examined tissue sections
+ +	Moderate lesion recorded in 16–35% of examined tissue sections
+ + +	Sever lesion recorded in more than 35% of examined tissue sections

#### Statistical analysis

The data were presented as mean ± SD. Parametric data were analyzed using one-way analysis of variance (ANOVA), followed by the Tukey post hoc test for multiple comparisons. However, analyzing more than one variable as in PASI score, two-way ANOVA followed by a Tukey post hoc test was conducted to assess both factors; time and treatment. Statistical analysis was performed using Prism software (version 8; GraphPad Software, Inc., CA, USA). The significance level for all comparisons was set at *p* < 0.05.

## Results

### Anti-psoriasis activity

#### Effects of *A*. *chinensis* flower and stalk as well as *A*. *squamatus* extract treatment on IMQ-induced psoriasis-like symptoms

**Fig. 1:**
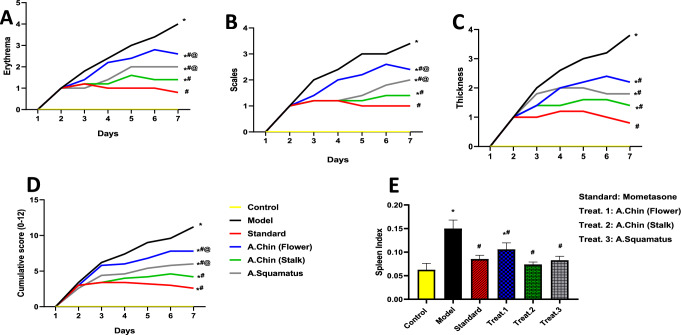
PASI score and spleen index of *A. chinensis flower and stalk as well as A. squamatus* extracts treatment in BALB/c mice subjected to IMQ-induced psoriasis. Photomicrographs represent skin erythema [A], scaling [B], epidermal thickness [C], cumulative score [D] and spleen index [E]. All data were expressed as mean ± SD (*n* = 5/group), using two-way ANOVA followed by Tukey’s post hoc test for PASI score and one-way ANOVA followed by Tukey’s post hoc test for spleen index; *p* < 0.05. * vs control group, ^#^ vs IMQ group, ^@^ vs mometasone group

Application of IMQ cream for seven consecutive days worsened epidermal erythema (score 4; Fig. 1A), scaling (score 3.4; Fig. 1B) and thickening (score 3.8; Fig. 1C), compared to normal skin score 0), as indicated by PASI scores. Conversely, oral treatment with *A. chinensis flower* and *stalk* as well as *A. Squamatus extracts* succeeded to reduce these psoriasis-like symptoms. As illustrated in Fig. 1, these three oral treatments notably improved skin redness, reduced scaling and decreased epidermal thickness as compared to the insult group. Additionally, the effectiveness of *A. chinensis* stalk treatment was comparable to the standard mometasone treatment with no significant statistical difference meanwhile, treatment with *A. chinensis* flower and *A. Squamatus* extracts displayed statistical significance compared to mometasone group.

**Fig. 2:**
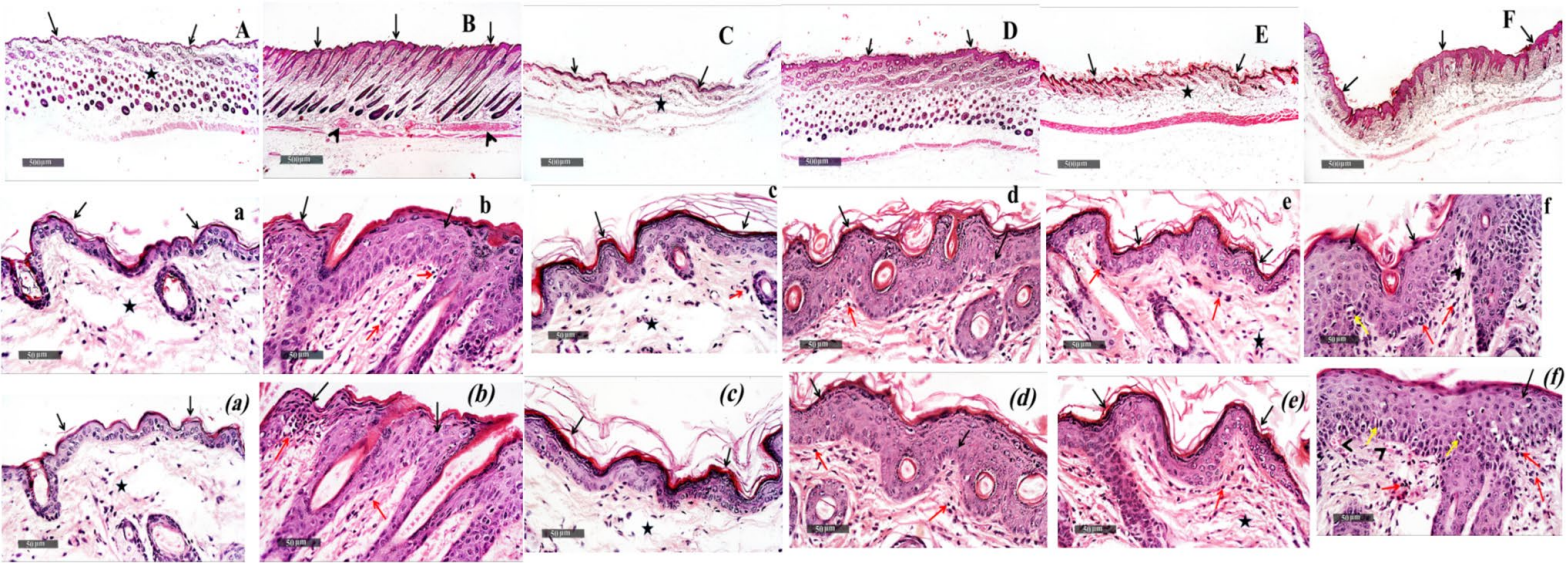
Histological evaluation of *A.chinensis* flower and stalk as well as *A.squamatus* extracts treatment in BALB/c mice subjected to IMQ-induced psoriasis. Photomicrographs depicting H&E staining of the control group [A, a, (a)], the IMQ-group [B, b, (b)], the mometasone group [C, c, (c)], the *A.squamatus* group [D, d, (d)], the *A.chinensis* stalk group [E, e, (e)], and the *A.chinensis* flower group [F, f, (f)]. Scale bar = 50 μm. Black arrow indicated the epidermal layer of the skin, black star highlighted the undamaged dermal layer characterized by well-arranged collagen fibers and hair follicles, arrow ahead pointed to congested and dilated blood vessels beneath the epidermis, red arrow denoted the infiltration of inflammatory cells, and yellow arrow signified localized vacuolization observed in the epidermal keratinocytes

The spleen index serves as a marker for immune system activity, reflecting the extent of lymphocyte proliferation in mice. In the present investigation, topical application of IMQ cream portrayed substantial increase in the spleen index (*F*_(5, 24)_ = 33.65, *p* < 0.0001) by 1.5-folds, compared to control group (Fig. 1E). In opposition, oral treatment with *A. chinensis flower* and *stalk* as well as *A. Squamatus* extracts resulted in decreases in the spleen index by 30, 51 and 45%, respectively, as compared to the IMQ-group. Indeed, the spleen index of *A. chinensis stalk and A. squamatus* showed no significant difference when compared to mometasone group. However, significant differences were observed in the spleen index of groups treated with *A. chinensis flower* when compared to the standard treatment.

Based on PASI score and spleen index, *A. chinensis stalk extract* appears to more effective than extracts of *A. chinensis flower* and *A. Squamatus* in mitigating IMQ-Induced psoriasis-like symptoms.

#### Effects of *A*. *chinensis* flower and stalk as well as *A*. *squamatus* extracts treatment on IMQ-induced histopathological alterations

As depicted in Fig. 2, histological analysis of control samples revealed normal skin architecture, with thin epidermis containing intact keratinocytes (black arrow), a well-organized dermal layer with collagen fibers and hair follicles (black star) and intact subcutaneous tissue. However, application of IMQ cream resulted in a significant increase in epidermal thickness (Acanthosis) across all skin samples (black arrow), accompanied by mild hyperkeratosis, slight clubbing of rete ridges, and micro-abscess formation. In parallel, dermal blood vessels appeared congested and dilated (arrowhead), with moderate infiltration of inflammatory cells throughout the dermal layer (red arrow). Conversely, treatment with mometasone showed preserved histological features, including minimal epidermal thickening (black arrow), intact dermal (star) and subcutaneous layers, and minimal inflammatory cell infiltration (red arrow) compared to all other treatment groups.

Oral Administration of *A. chinensis flower* and *stalk* as well as *A. squamatus* extracts were evaluated in a psoriasis model induced by IMQ in BALB/c mice. Mice treated with *A. chinensis* flower extract displayed similar pathological characteristics to the diseased samples including, multiple focal areas of degenerated basal cell layer of dermis (yellow arrow), along with focal intraepidermal inflammatory cells infiltrates. In contrast, mice treated with *A. chinensis* stalk extract exhibited more organized morphological features of the skin layers with a notable reduction in epidermal thickness and an almost intact dermal layer, resembling the effect of standard treatment, mometasone. Furthermore, mice treated with *A. squamatus* extract showed moderate protective effects on epidermal thickness, with visibly intact basal layer cells (black arrow), and a significant decrease of dermal inflammatory changes (red arrow). Severity scores of skin histopathological alterations were displayed in (Table 3).

**Table 3:** Histological assessment of skin lesions after treatment with *A. chinensis flower and stalk and well as A. squamatus in* BALB/c mice subjected to IMQ-induced psoriasis

	Control	IMQ	Mometasone	*A. chinensis* Flower	*A. chinensis* stalk	*A. squamatus*
Epidermal thickening	−	+ + +	−	+ + +	+	+ +
Dermal inflammatory cell infiltrates	−	+ + +	+	+ +	+	+
Congested blood vessels	−	+ +	−	+	−	−

#### Effect of *A*. *chinensis* stalk fractions on IMQ-induced psoriasis-like symptoms

**Fig. 3:**
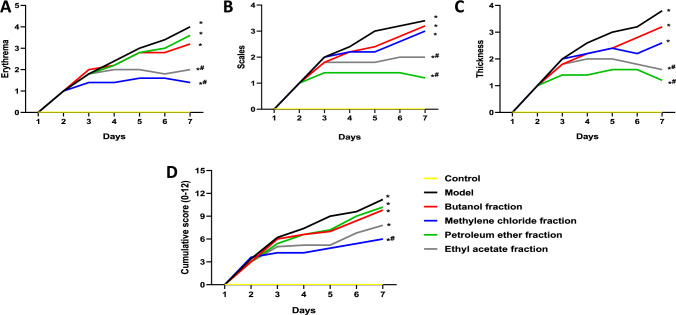
PASI score of butanol, methylene chloride, petroleum ether and ethyl acetate fractions of *A. chinensis stalk* treatment in BALB/c mice subjected to IMQ-induced psoriasis. Photomicrographs represent skin erythema [A], scaling [B], epidermal thickness [C], and cumulative score [D]. All data were expressed as mean ± SD (*n* = 5/group), using two-way ANOVA followed by Tukey’s post hoc test; *p* < 0.05. * vs control group, ^#^ vs IMQ group

The topical application of IMQ cream over the course of a week exacerbated epidermal erythema (score 4; Fig. 3A), scaling (score 3.4; Fig. 3B) and thickening (score 3.8; Fig. 3C) when compared to the control group, as evidenced by the increase in PASI scores. Conversely, topical application of *methylene chloride* and* ethyl acetate* fractions of *A. chinensis* stalk effectively alleviated these psoriasis-like symptoms, via reducing epidermal erythema (score 1.4 and 2), scaling (score 1.2 and 2), and thickness (1.2 and 1.6) in comparison with the insult group. However, topical application of *butanol* and *petroleum ether* fractions of *A. chinensis* stalk failed to ameliorate psoriasis like pathological features. Notably, treatment with *methylene chloride* fractions of *A. chinensis* stalk resulted in the most substantial decrease in cumulative PASI score compared to all other treatments.

#### Effect of *A*. *chinensis* stalk fractions on IMQ-induced histopathological impairment

Examination of control samples revealed the presence of normal, well-organized histological features in the skin layers. The epidermal layer appeared thin with intact keratinocytes *(black arrow)*. Additionally, the dermal layer displayed well-organized collagen fibers and healthy hair follicles (black star), devoid of abnormal inflammatory cell infiltration, and the subcutaneous tissue remained intact. In contrast, the application of IMQ cream resulted in a notable increase in epidermal thickness, known as acanthosis, observed throughout the skin tissue sections (black arrow). This was accompanied by significant degenerative changes in the basal cell layer (arrowhead). In parallel, inflammatory cell infiltration was markedly evident throughout the dermal layer (red arrow), with intradermal lymphocytic infiltrates and congested, dilated deep dermal blood vessels observed.

**Fig. 4:**
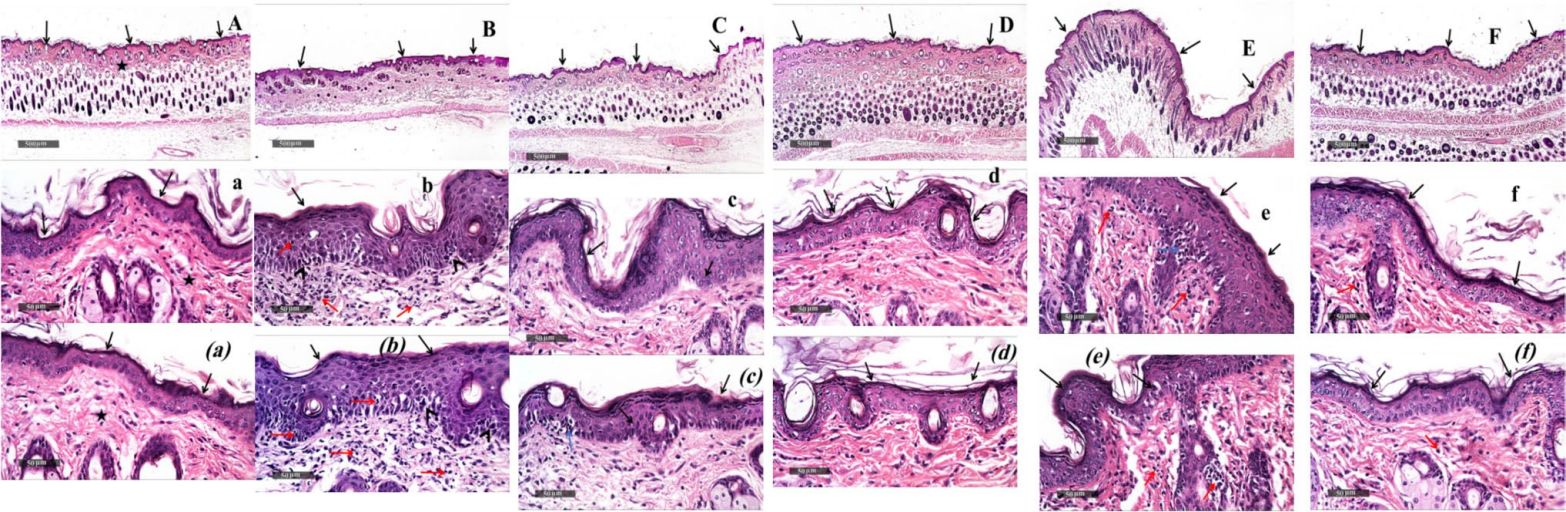
Histological investigation of butanol, methylene chloride, petroleum ether and ethyl acetate fractions of *A*. *chinensis* stalk treatment in BALB/c mice subjected to IMQ-induced psoriasis. Photomicrographs depicting H&E staining of the control group [A, a, (a)], the IMQ-group [B, b, (b)], the butanol fraction group [C, c, (c)], the methylene chloride fraction group [D, d, (d)], the petroleum ether fraction group [E, e, (e)], and ethyl acetate fraction group [F, f, (f)]. Scale bar = 50 μm. Black arrow indicated the epidermal layer of the skin, black star highlighted the undamaged dermal layer characterized by well-arranged collagen fibers and hair follicles, arrow ahead pointed to degenerative changes of the basal cell layer, red arrow denoted the infiltration of inflammatory cells, and blue arrow signified localized micro-abscess observed in the epidermal layer

The topical application of *A.chinese* stalk fractions were investigated in BALB/c mice using IMQ-induced psoriasis model. *Butanol fraction* exhibited mild improvement in epidermal layer thickening *(black arrow), along* with multiple records of micro-abscess *(blue arrow).* However, there was a noteworthy reduction in dermal inflammatory cell infiltrates. On the other hand, *Methylene chloride fraction* displayed the well-organized morphological features of skin layers across all groups, accompanied by a significant decrease in epidermal thickness and a down regulation of abnormal inflammatory changes. *Petroleum ether fraction* exhibited similar characteristics to the butanol fraction samples, but with higher levels of dermal inflammatory cell infiltrates *(red arrow).* Finally, *the Ethyl acetate fraction* showed significant improvement in the morphological features of skin layers similar to methylene chloride fraction. Nonetheless, there were occasional mild focal accumulations of dermal inflammatory cells* (red arrow),* along with mild congestion in deep dermal blood vessels (Fig. 4).

*Based on the results of PASI score, and histopathological examination, the methylene chloride fraction of A. chinensis stalk was chosen for further exploration to elucidate the biochemical mechanisms driving its anti-psoriasis effects.*

#### Methylene chloride fraction of *A*. *chinensis* stalk mitigated IMQ-induced inflammatory response

**Fig. 5:**
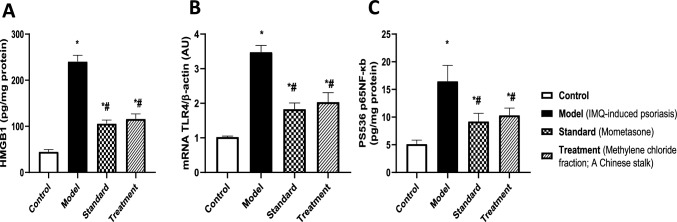
Effect of treatment with methylene chloride fraction of *A. chinensis* stalk on [A] HMGB1 content, [B] mRNA TLR4 and [C] pS536 NFκB p65 content in BALB/c mice subjected to IMQ-induced psoriasis. Data were presented as mean ± SD (*n* = 6/group), using one-way ANOVA followed by Tukey’s post hoc test; *p* < 0.05. * Vs control group, ^#^ vs IMQ group. HMGB1; High mobility group box1, TLR4; Toll-like receptor 4, pS536 NFκB p65; Phospho-serine 536 nuclear factor Kappa B p65

Topical application of IMQ provoked a notable increase in HMGB1 content (A), as well as the mRNA expression of TLR4 (B) and pS536-NFκB p65 content (C) by 4.3-, 2.4- and 2.2-folds, respectively, as compared to control group (Fig. 5). On the other hand, treatment with methylene chloride fraction of *A. chinensis* stalk effectively reduced inflammatory response, resulting in decreased HMGB1 content by 52%, TLR4 gene expression by 42%, and p-NFκB content by 37%, relative to the insult group. Moreover, the effectiveness of methylene chloride fraction was comparable to the standard treatment, mometasone.

**Fig. 6:**
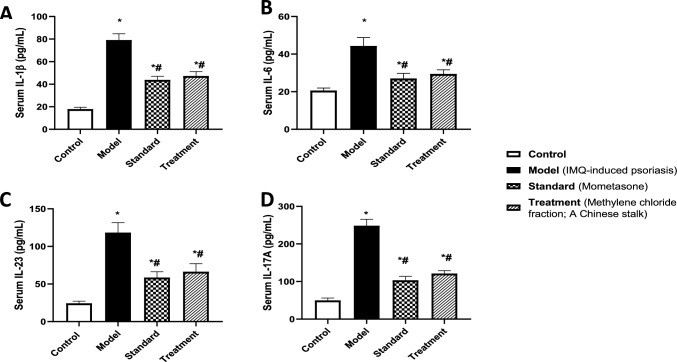
Effect of treatment with methylene chloride fraction of *A. chinensis* stalk on serum [A] IL-1β, [B] IL-6, [C] IL-23 and [D] IL-17A levels in BALB/c mice subjected to IMQ-induced psoriasis. Data were presented as mean ± SD (*n* = 6/group), using one-way ANOVA followed by Tukey’s post hoc test; *p* < 0.05. * Vs control group, ^#^ vs IMQ group. IL-1β; Interleukin 1 beta, IL-6; Interleukin 6, IL-23; Interleukin 23, IL-17A; Interleukin 17 A

In parallel, the application of IMQ topically led to massive production of pro-inflammatory cytokines, exacerbating the inflammatory condition. Indeed, IMQ-mice displayed an upsurge in serum levels of IL-1β (A), IL-6 (B), IL-23 (C), and IL-17 (D) by 3.4-, 1.2-, 3.9- and 4-folds, respectively, as compared to control (Fig. 6). On the contrary, treatment with methylene chloride fraction of *A. chinensis* stalk succeeded to mitigate inflammatory response via reducing IL-1β, IL-6, IL-23, and IL-17 levels by 40, 34, 44, and 51%, respectively, relative to IMQ-induced psoriasis group. Additionally, the effects of methylene chloride fraction are comparable to the mometasone group.

#### Methylene chloride fraction of *A*. *chinensis* stalk attenuated IMQ-induced oxidative stress status

**Fig. 7:**
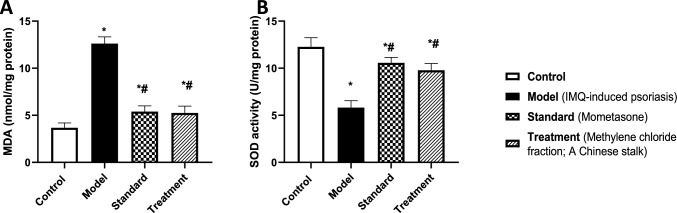
Effect of treatment with methylene chloride fraction of *A. chinensis* stalk on [A] MDA content and [B] SOD activity in BALB/c mice subjected to IMQ-induced psoriasis. Data were presented as mean ± SD (*n* = 6/group), using one-way ANOVA followed by Tukey’s post hoc test; *p* < 0.05. * Vs control group, ^#^ vs IMQ group. MDA; Malondialdehyde, SOD; Superoxide dismutase

As depicted in Fig. 7, exposure to IMQ for 7 consecutive days induced an oxidative stress status that was evidenced by a substantial increase in lipid peroxidation biomarker, MDA content (A) (3.4-folds) coupled with a decline in antioxidant defense mechanism, SOD activity (B) (47%), compared to the control group. In opposition, treatment with methylene chloride fraction of *A. chinensis* stalk attenuated oxidative stress status via reducing the elevated MDA content by 58% along with enhancing SOD activity by 68%, relative to the insult group. Notably, the result of methylene chloride fraction was comparable to the standard treatment, showing no statistical significance.

### Phytochemical composition and chemical analysis

**Fig. 8:**
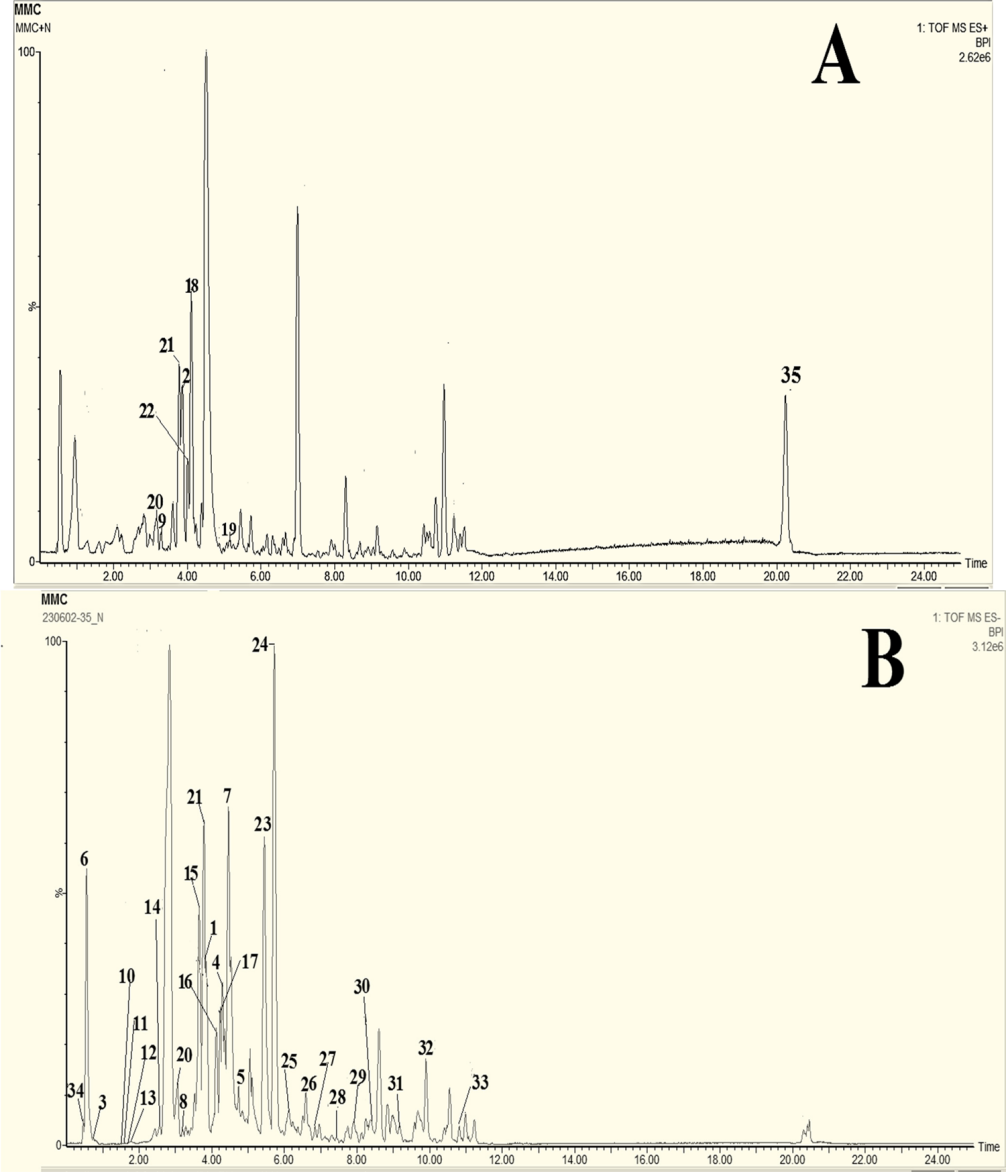
Representative *UHPLC-MS/MS* of Methylene chloride fraction of the stalks of *A. chinensis*. (A): the methylene chloride fraction of the stalks of *A. chinensis* was analyzed in positive ion mode, (B): the methylene chloride fraction of the stalks of *A.chinensis* was analyzed in negative ion mode Assigned peaks numbers follow those listed in (Table 4) for metabolite identification using *UHPLC-MS/MS*

The methylene chloride fraction of *A. chinensis* stalks demonstrated superior anti-psoriatic efficacy compared to other fractions. Subsequently, it was analyzed using a high-resolution UHPLC-MS/MS technique in both positive and negative ion modes (Fig. 8). Compared to the positive-ion mode, the MS spectra of the negative-ion mode exposed better sensitivity and more visible peaks. A total of 35 metabolites. were tentatively elucidated by comparing their retention times, and MS data (accurate mass, and fragmentation pattern in negative ion mode or positive ion modes) of the compounds detected with compounds reported in the literature and searching in public online databases all data are presented in Table 4, and supplementary material (Online Resource). The identified metabolites were classified as 2 flavonoids, 3 dicarboxylic acids, 10 phenolic acids, 2 lignans, 1 monoterpenoid hydroxylactone, 1 benzofuran, 3 coumarins, 11 fatty acids, 1 sugar, and 1 amino acid. It is noteworthy that, phenolic compounds represent the predominant chemical constituents within the methylene chloride fraction isolated from *A. chinensis* stalks.

**Table 4:** Peak assignments of metabolites in methylene chloride fraction of stalk of *A.chinensis* using *UHPLC-MS/MS* in negative/positive ion modes

Peak No	Rt (min)	Identified Compounds	Exact M.F	Exact Molecular mass	[M-H]^−^	[M + H]^+^	MS^2^ fragment [M-H]^−^	MS^2^ fragment [M + H]^+^	Error (ppm)	References
Flavonoids										
1	3.84	Myricitrin	C_21_H_20_O_12_	464.38	463.0887	–	317,151	–	2.2	Peixoto Araujo et al. (2020), Sun et al. (2018)
2	3.84	Hydroxyluteolin	C_15_H_10_O_7_	302.24	–	303.0517	–	285,169,135,123,95	4	Fu et al. (2020)
Dicarboxylic acids and derivatives										
3	0.79	Citramalate	C_5_H_8_O_5_	147.0295	–	148.11	129,87,85	–	1.4	Liu et al. (2021)
4	4.29	Nonanedioic acid (Azelaic acid)	C_9_H_16_O_4_	188.22	187.0975	–	169,125,97	–	2.7	Bondia‐Pons et al. (2013)
5	4.87	Sebacic acid	C_10_H_18_O_4_	202.25	201.1134	–	183,139,111	–	3.5	Liu et al. (2021)
Phenolic compounds										
6	0.55	Caffeoyl-*O*-hexoside derivative	C_18_H_18_O_9_	378.33	377.0858	–	341,179,89	–	–3.86	Tóth et al. (2015)
7	4.46	Ferulic acid	C_10_H_10_O_4_	194.18	193.0509	–	149, 137,134	–	4.1	Zengin et al. (2018), Zheleva-Dimitrova et al. (2020)
8	3.18	Caffeoylquinic acid	C_16_H_18_O_9_	354.31	353.0872	–	191,179,135	–	−0.16	Karaköse et al. (2011)
9	3.42	Paeonol	C_9_H_10_O_3_	166.17	–	167.0713	–	149,121,91, 77	2.88	Sun et al. (2018)
10	1.51	Hydroxyhexosylbenzoic acid	C_13_H_16_O_9_	316.26	315.0719	–	153,109	–	1	Jaiswal et al. (2014)
11	1.61	Dihydroxybenzoic acid I	C_7_H_6_O_4_	154.12	153.0188	–	109	–	0.0	Jaiswal et al. (2014), Ostrowski et al. (2014), Zheleva-Dimitrova et al. (2020)
12	1.77	Dihydroxybenzoic acid II (Protocatechuic acid)	C_7_H_6_O_4_	154.12	153.0186	–	109	–	−1.3	
13	1.84	Dihydroxybenzoic acid III ( Gentisic acid)	C_7_H_6_O_4_	154.12	153.0193	–	109	–	3.3	
14	2.57	Hydroxy benzoic acid	C_7_H_6_O_3_	138.12	137.0236	–	93	–	−2.2	Jaiswal et al. (2014), Ostrowski et al. (2014)
15	3.65	Vanillic acid	C_8_H_8_O_4_	168.15	167.0348	–	152,108	–	2.4	Liu et al. (2021), Ostrowski et al. (2014)
Lignans										
16	4.06	Pinoresinol *O*-*β*-d-hexopyranoside	C_26_H_32_O_11_	520.53	519.1877	–	357,151,136	–	2.05	Zhang et al. (2020)
17	4.11	Syringaresinol hexoside	C_28_H_36_O_13_	580.58	579.2091	–	417,387,181,151	–	2.2	Steingass et al. (2015)
Monoterpenoid hydroxylactone										
18	4.11	Loliolide	C_11_H_16_O_3_	196.24	–	197.1186	–	179,135,133,107	4.1	El Sayed et al. (2020)
Benzofuran										
19	5.13	Licocoumarone	C_20_H_20_O_5_	340.37	–	341.1412	–	323,271,137	6.7	Emad et al. (2022)
Coumarin										
20	3.07	Dihydroxy coumarin (Esculetin)	C_9_H_6_O_4_	178.14	177.0191	179.0347	149,133,121,105,89	151,123	1.7	Sun et al. (2018), Tóth et al. (2015)
21	3.77	Hydroxycoumarin (Umbelliferone)	C_9_H_6_O_3_	162.14	161.0243	163.0402	133	119,107,91 ,77 –	2.5	Bhargav et al. (2018), Sun et al. (2018)
22	3.87	Scopoletin	C_10_H_8_O_4_	192.17	–	193.0509	–	161,137,133,105	4.1	Cannavacciuolo et al. (2023), Sun et al. (2018)
Fatty acids										
23	5.45	Trihydroxyoctadecadienoic acid	C_18_H_32_O_5_	328.23	327.2194	–	229	–	7	Peixoto Araujo et al. (2020)
24	5.71	Pinellic acid	C_18_H_34_O_5_	330.46	329.2362	–	229, 211,183,171	–	10.3	Nadeem et al. (2020)
25	6.13	Tianshic acid	C_18_H_34_O_5_	330.46	329.2361	–	171	–	10	Liu et al. (2021)
26	6.52	Trihydroxynonadecenoic acid	C_19_H_36_O_5_	344.49	343.2515	–	171	–	9	Zheleva-Dimitrova et al. (2020)
27	6.77	Trihydroxyoctadecenoic acid	C_18_H_34_O_5_	330.46	329.2361	–	311,293,211,171	–	10	Zheleva-Dimitrova et al. (2020)
28	7.42	Dihydroxyoctadecenoic acid	C_18_H_34_O_4_	314.46	313.2406	–	295,201,171	–	8.6	Zheleva-Dimitrova et al. (2020)
29	7.91	Hydroxyoctadecatrienoic acid	C_18_H_30_O_3_	294.43	293.2143	–	275,235,183, 171	–	8.9	Zheleva-Dimitrova et al. (2020)
30	8.40	Hydroxyoctadecadienoic acid	C_18_H_32_O_3_	296.45	295.2297	–	277,171,113	–	8.1	Nadeem et al. (2020)
31	9.33	Palmitic acid (Cetylic acid)	C_16_H_32_O_2_	256.42	255.2339	–	59,44	–	5.86	Made Ratih et al. (2019)
32	9.90	Hydroxyhexadecanoic acid (Juniperic acid)	C_16_H_32_O_3_	272.42	271.2281	–	225,145,59, 44	–	2.9	Zhang et al. (2023), Made Ratih et al. (2019)
33	10.89	Hydroxy stearic acid	C_18_H_36_O_3_	300.48	299.2582	–	255	–	−1.3	Peixoto Araujo et al. (2020)
Sugar										
34	0.51	Sucrose	C_12_H_22_O_11_	342.30	341.1086	–	89,59	–	0.6	Liu et al. (2021)
Amino acid										
35	20.24	Proline	C_5_H_9_NO_2_	115.13	–	116.0712	–	70,68	0.0	Zengin et al. (2018)

#### Identification of phenolic compounds

The phenolic compounds are considered the major group in the methylene chloride fraction of the stalk of *A. chinensis*. Phenolic compounds are a heterogeneous group of secondary metabolites generated during plant metabolism. The chemical structure of phenolic compounds was characterized by an aromatic ring with one or more hydroxyl group's substitution (García-Perez et al. 2017). Polyphenols are the most common antioxidants found in fruit and vegetable-based diets. Some phenolic compounds can also inhibit pro-inflammatory mediator’s activity or gene expression, such as cyclooxygenase (COX). Phenolic compounds can also up- or down regulate transcriptional elements involved in antioxidant pathways, such as nuclear factor-κB (NF-κB), these compounds believed to reduce oxidative stress either individually or in combination (Contardi et al. 2021).

##### Flavanoids

Flavonoids have been frequently reported from *A. chinensis* as sugar conjugates, in general as *O*-glycosides (Bhargav et al. 2018). In MS/MS analysis, two flavone peaks were detected, the nature of the sugars could be revealed by the elimination of the sugar residue, that may be 162 amu (hexose; glucose or galactose), 132 amu (pentose; arabinose), and 146 amu (deoxysugar; rhamnose). Peak no. 1 (negative ion mode, Rt = 3.84 min) was characterized by a deprotonated molecular ion at *m*/*z* 463.0887 [M-H]^−^ with exact molecular formula C_21_H_20_O_12_. The base peak fragment ion was detected at *m*/*z* 317, corresponding to myricetin aglycon which derived from the loss of rhamnose moiety [M-H-146]^−^ and by comparing the data with the reported literature, it could be tentatively identified as is myricetin rhamnoside (Peixoto Araujo et al. 2020; Sun et al. 2018). Furthermore, the predominant flavonoid of the flower extract of* A.chinensis* was myricetin and myricetin-3-*O*-rhamnoside (Bhargav et al. 2018). While peak no. 2 (positive ion mode, Rt = 3.84) afforded [M + H]^+^ ion at *m*/*z* 303.0517 with exact molecular formula C_15_H_10_O_7_, which referred to penta hydroxyl substitution flavone derivative. The diagnostic ion at *m*/*z* 285 indicating loss 18 amu (H_2_O). Cleavage of the 1 and 3 bonds in flavone gives rise to two product ions, ^1,3^A^+^ ion at *m*/*z* 169 and ^1,3^B^+^ ion at *m*/*z* 135. This product ion pair clearly indicates the substitutions in the 3 OH and 2 OH on A and B rings, respectively. A further fragmentation by losses of small molecules; *m*/*z* 123 (the base peak) can be generated from *m*/*z* 169 (^1,3^A^+^) by successive losses of 18 amu (H_2_O) (Fu et al. 2020), and by comparing the data with the available literature, compound (2) is tentatively identified as Hydroxyluteolin. However, luteolin was isolated from ethyl acetate fraction of *A.chinensis* of flower petals extract (Bhargav et al. 2018).

##### Identification of phenolic acids

Phenolic acids and their derivatives exhibit strong antioxidant properties, helping to reduce oxidative stress as well as inflammation in the body, which can lead to chronic diseases (García-Perez et al. 2017). Hydroxybenzoic (C1–C6) and hydroxycinnamic (C3–C6) acids are potent, and highly abundant phenolic compounds in various herbs and spices that offer unique health benefits (Devkota and Aftab 2022; Cáceres-Velez et al. 2022). Moreover, hydroxybenzoic acids and hydroxycinnamic acids have anti-inflammatory effects, which may help to further decreasing the chance of developing chronic illnesses (Cáceres-Velez et al. 2022). MS/MS full scan revealed the detection of ten phenolic acids classified as: two hydroxyl cinnamic acids peaks (6, 7), one monoacyl quinic acid identified as peak (8), one phenol assigned as peak (9), and six hydroxyl benzoic acid referred as peaks from (10 to 15) were reported in methylene chloride fraction of *A.chinensis* of stalks.

A characteristic fragmentation pattern was observed for hydroxycinnamic acid derivatives in MS/MS spectra, with diagnostic ions at *m*/*z* 191 for quinic acid, 179 for caffeic acid, and 193 for ferulic acid. In peak no. 6 (negative ion mode, Rt = 0.55min) was characterized by a deprotonated molecular ion [M-H]^−^ at *m*/*z* 377.0858. In MS/MS analysis, the fragment ion at *m*/*z* 341 [M-H- 2H_2_O]^−^, and fragment ion at *m*/*z* 179 [M-H-162]^−^ indicating the presence of caffeic acid moiety, which isolated from *A.squamatus* (Sperotto et al. 2002), and corresponding to loss of one hexose unit. By comparing the fragmentation pattern with the literature found it was caffeoyl-*O*-hexoside derivative with exact molecular formula C_18_H_18_O_9_ (Tóth et al. 2015; El-Hawary et al. 2020). Peak no. 7 (negative ion mode, Rt = 4.46min) was characterized by a deprotonated molecular ion [M-H]^−^ at *m*/*z* 193.0509. Its MS/MS fragmentation pattern has been reported to involve the releasing of carboxyl group detected at fragment ion *m*/*z* 149[M-H-COO]^−^, followed by releasing of methyl group at *m*/*z* 134 [M-H-COO-CH_3_]^−^. This pattern of fragmentation was characteristic for ferulic acid, which isolated from *A. tataricus* (Yang et al. 2015a), with the exact molecular formula C_10_H_10_O_4_. By collecting the data and comparing it with literature, it was determined as ferulic acid (Zheleva-Dimitrova et al. 2020; Zengin et al. 2018; Abdou et al. 2021). Peak no 8 (negative ion mode, Rt = 3.184 min), was characterized by a deprotonated molecular ion [M-H]^−^ at *m*/*z *353.0872 with exact molecular formula C_16_H_18_O_9_. Its MS/MS fragmentation pattern has been reported to involve releasing of both quinic acid moiety at* m*/*z* 191, and the fragment ion at *m*/*z* 179 indicated to release of caffeic acid moiety, and the fragment ion at *m*/*z* 135 [M-H-quinic–caffeic-COO]^−^ referring to the release of carbon dioxide molecule. On comparison with the literature, it was tentatively identified as caffeoylquinic acid (Karaköse et al. 2011), and it isolated from *A.tataricus* (Zhao et al. 2015). Indeed, the presence of the fragment ion at *m*/*z* 109 in the MS spectra is diagnostic for [M-H-dihydroxyl benzoic acid]^−^, and the fragmentation of hydroxyl benzoic acids was characteristic by losing CO_2_ due to the presence of carboxyl group (Zheleva-Dimitrova et al. 2020). The deprotonated molecular ion [M-H]^−^ of peak no 10 (negative ion mode, Rt = 1.51 min) at *m*/*z* 315.0719. Its MS/MS fragmentation pattern has been reported to involve releasing of hexose sugar moiety [M-H-162]^−^ that was observed at *m*/*z* 153. The fragment ion at *m*/*z* 109 [M-H-C_6_H_12_O_6_-COO] referring to the loss of CO_2_ moiety, which was characteristic for dihydroxy benzoic acid (Zheleva-Dimitrova et al. 2020; Tóth et al. 2015), and by comparing this data with literature found that it was identified as hydroxyhexosylbenzoic acid, isolated from *A. himalaicus* (Xie et al. 2010), with exact molecular formula C_13_H_16_O_9_ (Jaiswal et al. 2014). The deprotonated molecular ions [M-H]^−^ of peaks no 11, 12, 13 (negative ion mode, Rt = 1.61,1.77, 1.84 min, respectively) were detected at *m*/*z* 153.0188, 153.0186, and 153.0193, respectively, and appeared in MS/MS spectra as three separated peaks with the same exact molecular formula C_7_H_6_O_4._ The base peak of all of these was observed at *m*/*z* 109 [M-H-COO]^−^, indicating that the loss of CO_2_, and these fragmentation was characteristic for dihydroxy benzoic acid. The three dihydroxy benzoic acid compounds were isolated from *A. himalaicus* (Xie et al. 2010). By comparing the data with the available literature, it was found that the three compounds were identified as dihydroxy benzoic acid (Jaiswal et al. 2014; Zheleva-Dimitrova et al. 2020; Ostrowski et al. 2014). The deprotonated molecular ion [M-H]^−^ of peak no. 14 (negative ion mode, Rt = 2.575 min) at *m*/*z* 137.0236 with exact molecular formula C_7_H_6_O_3_. The appearance of the fragment ion at *m*/*z* 93 referred to decarboxylation of structure [M-H-COO]^−^, via the cleavage of C-aromatic ring bond and losing of CO_2_ [M-H- 44]^−^. By comparing the data with the available literature, it was found that hydroxyl benzoic acid, isolated from *A. himalaicus* (Xie et al. 2010; Jaiswal et al. 2014; Ostrowski et al. 2014). The deprotonated molecular ion of peak no 15 at [M-H]^−^ *m*/*z* 167.0348 (negative ion mode, Rt = 3.654 min) was determined as vanillic acid, that isolated from *A. squamatus* (Sperotto et al. 2002), with the exact molecular formula C_8_H_8_O_4_. Its MS/MS fragmentation pattern has been reported to produce fragment ion at *m*/*z* 152 indicated the demethylation of the compound [M-H-CH_3_]^−^, and detected the fragment ion at *m*/*z* 108 referred to the decarboxylation of structure after releasing methyl moiety [M-H-CH_3_-COO]^−^ (Abdou et al. 2021).

#### Identification of dicarboxylic acids and derivatives

Three dicarboxylic acids were detected in full scan of MS/MS spectra represented as peaks no. 3, 4, and 5. The sequences of the fragmentation which was characteristic for dicarboxylic acid began with losing 18 amu [M-H-H_2_O]^−^, then decarboxylation by releasing 44 amu [M-H-CO_2_]^−^, and finally releasing 28 amu [M-H-CO]^−^ (Liu et al. 2021; Bondia-Pons et al. 2013). The deprotonated molecular ion formula of peaks no. 4 (negative ion mode, Rt = 4.29 min) [M-H]^−^ was detected at *m*/*z* 187.0975 with exact molecular formula C_9_H_16_O_4_. Its MS/MS fragmentation pattern has been reported to involve the releasing of water molecule from the structure leads to the appearance of the fragment ion at *m*/*z* 169 [M-H-H_2_O]^−^. Also, the decarboxylation was also detected in spectra at fragment ion *m*/*z* 125 [M-H-H_2_O-CO_2_]^−^. Then, the releasing of carbon monoxide moiety can be detected at fragment ion *m*/*z* 97 [M-H-H_2_O-CO_2_-CO]^−^. The releasing of two carboxylic groups indicates the structure relating to the dicarboxylic acid class. By comparing the data with the literature, it was identified as nonanedioic acid (azelaic acid) (Bondia‐Pons et al. 2013). The deprotonated molecular ion of peak no. 5 (negative ion mode, Rt = 4.87 min) [M-H]^−^ at *m*/*z* 201.1134 with exact molecular formula C_10_H_18_O_4_. Its MS/MS fragmentation pattern has been reported to involve producing the fragment ion at *m*/*z* 183[M-H-H_2_O]^−^, indicating a release of 18 amu from the structure, followed by decarboxylation and losing of 44 amu, which appeared clearly at *m*/*z* 139 [M-H-H_2_O-CO_2_]^−^, and finally releasing carbon mono-oxide at *m*/*z* 111 [M-H-H_2_O-CO_2_-CO]^−^. By comparing the fragmentation with the available literature, it was found that peak no. 5 may be identified as sebacic acid (Liu et al. 2021).

#### Identification of lignans

**Fig. 9:**
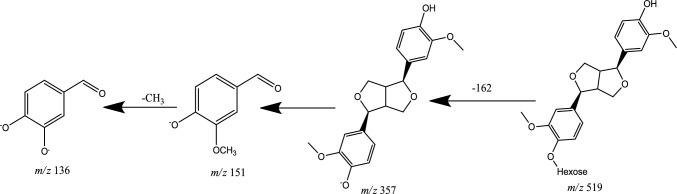
Fragmentation pattern of Pinoresinol hexopyranoside

Lignans are phytoestrogens that widely exist in nature, consisting of phenylpropanoid units (C6–C3), mainly in forming dimers, linked by the central C-atom, C (8), of their side chains (Steingass et al. 2015). Lignans are classified in six subgroups based upon the way in which the O-atom is incorporated in the skeleton and cyclization pattern: dibenzylbutanes, dibenzylbutyrolactones, arylnaphthalenes, terahydrofurans, furfurans, and dibenzocyclooctadienes (Zhang et al. 2014, 2020). In MS/MS spectra, two phenolic lignans belonging to furfuran class of lignans were detected in negative ion mode (Peak no. 16, 17). The characteristic fragmentation patterns of furfuran lignans in negative ion mode were the cleavage in the side chain at *α* and *β* position to produce the fragment ion at *m*/*z* 151 due to the release of guaiacyl moiety, and/or produce fragment ion at *m*/*z* 181 resulting from the releasing of syringyl moiety (Eklund et al. 2008). The deprotonated molecular ion [M-H]^−^ of peak no. 16 (negative ion mode, Rt = 4.06min) at *m*/*z* 519.1877 with exact molecular formula C_26_H_32_O_11_, produced the product ion at *m*/*z* 357 afforded deglycosidation by losing hexose sugar [M-H-162]^−^. The predominant ion at *m*/*z* 151 was specific for the releasing of guaiacyl molecule due to cleavage of the side chain between *α* and *β* position [M-H-C_6_H_12_O_6_-C_8_H_7_O_3_]^−^, and finally, the fragment ion at *m*/*z* 136 was referred to the demethylation [M-H-C_6_H_12_O_6_-C_8_H_7_O_3_-CH_3_]^−^. After comparing the fragmentation pattern with the literature, it was identified as pinoresinol *O*-*β*-d-hexopyranoside (Fig. 9) (Zhang et al. 2020), and it was isolated from *A. tataricus* (Su et al. 2019).

**Fig. 10:**
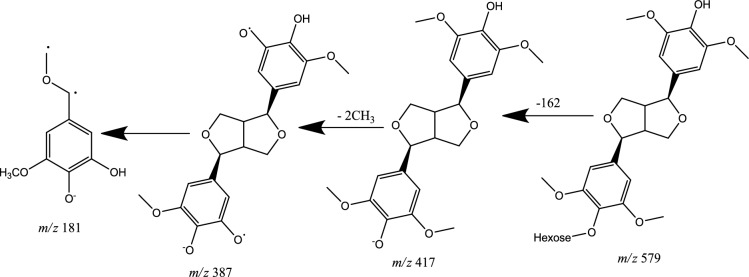
Fragmentation pattern of syringaresinol hexoside

The deprotonated molecular ion [M-H]^−^ of peak no. 17 (negative ion mode, Rt = 4.11min) at *m*/*z* 579.2091 with exact molecular formula C_28_H_36_O_13._ The deglycosidation of the structure produced at fragment ion *m*/*z* 417 by losing hexose sugar [M-H-162]^−^. Moreover, the fragment ion appeared at *m*/*z* 387 was due to the loss of two methyl radicals [M-H-162–30]^−^. However, the fragment ion at *m*/*z* 181 due to cleavage of the side chain between *α* and *β* position and releasing of syringyl radical [M-H-C_6_H_12_O_6_-2 CH_3_-C_9_H_10_O_3_]^−^; finally, the fragment ion at *m*/*z* 151 indicating the loss of another two methyl radical [M-H-C_6_H_12_O_6_-2 CH_3_-C_9_H_10_O_3_-2 CH_3_]^−^. By comparing the data with the literature, compound 17 was identified as syringaresinol hexoside (Fig. 10) (Steingass et al. 2015), and it was isolated from *A. tataricus* (Su et al. 2019).

#### Identification of monoterpenoid hydroxylactone

One monoterpenoid hydroxylactone was detected in MS/MS analysis positive ion mode and determined as loliolide, (peak 18), with exact molecular formula C_11_H_16_O_3_. The protonated molecular ion [M + H]^+^of peak no 18 (positive ion mode, Rt = 4.11 min) at *m*/*z* 197.1186. The predominant fragment at *m*/*z* 179 indicated the releasing of H_2_O from the lactone ring [M + H- H_2_O]^+^, the fragment ion at *m*/*z* 135 exposed the loss of 44amu [M + H- H_2_O-COO]^+^, and finally, the fragment ion at *m*/*z* 107 due to loss 28 amu and release C_2_H_4_ moiety [M + H- H_2_O-COO-C_2_H_4_]^+^ (Xi et al. 2021), and it was isolated from *A. sampsonii* (Gao 2010).

#### Identification of coumarin

Coumarins have the parent nucleus structure of benzopyrone. In general, the fragmentation pattern of such compounds is that loss of CO_2_ or CO from pyran-2-one moiety (Cannavacciuolo et al. 2023). Three coumarins have been tentatively identified in MS/MS spectra. The deprotonated molecular ion [M-H]^−^ of peak no. 20 (negative ion mode, Rt = 3.06min) at *m*/*z* 177.0191 with exact molecular formula C_9_H_6_O_4_. The [M-H]^–^ ion fragmented into the characteristic ions at *m*/*z* 149, 133, 121, and 105, corresponding to [M-H-CO]^–^, [M-H-CO_2_]^–^, [M-H-2CO]^–^, and [M-H-2CO-OH], respectively. By comparing fragmentation patterns with data in the literature, isolated from *A. yomena* (Kim et al. 2014), it could be identified as esculetin (Tóth et al. 2015; Sun et al. 2018). Peak no. 21 (positive ion mode, Rt = 3.77min) showed [M + H]^+^ ions at *m*/*z* 163.0402 with exact molecular formula (C_9_H_6_O_3_) and the obtained product ions at *m*/*z* 119 and 107 resulting from the progressive loss of a carbon dioxide [M + H-CO_2_]^+^ and two carbon monoxides[M + H-2CO]^+^ moieties, respectively. By comparing fragmentation patterns with data in the literature, it is tentatively identified as umbellifron, and it was isolated from *A.chinensis* (Cannavacciuolo et al. 2023; Sun et al. 2018; Bhargav et al. 2018). The protonated molecular ion [M + H]^+^ of peak no. 22 (positive ion mode, Rt = 3.87min) at *m*/*z* 193.0509 with exact molecular formula C_10_H_8_O_4_. The product ion spectra of [M + H]^+^ at *m*/*z* 193 was characterized firstly by the loss of CH_3_ and CO molecules. The [M + H]^+^ ion at *m*/*z* 193 lost CH_3_OH moiety to generate a product ion at *m*/*z* 161. Therefore, the representative fragment ions were [M + H-CH_3_OH]^+^ at *m*/*z* 161, [M + H-CH_3_OH-CO]^+^ at *m*/*z* 133, and [M + H-CH_3_OH-2CO]^+^ at *m*/*z* 105. By comparing fragmentation patterns with data in literature identified as scopoletin (Cannavacciuolo et al. 2023; Sun et al. 2018). It was isolated from *A. tataricus* (Ng et al. 2003).

#### Identification of fatty acids

Fragmentation of fatty acids was dominated by loss of H_2_O and carboxyl moieties. Negative ionization MS/MS analysis revealed numerous hydroxyl fatty acids as major peaks in the last part of the spectra. The several biological activities of hydroxyl fatty acids increased its importance which can be used as anti-inflammatory, antimicrobial, antifungal, and cytotoxic effects (Zheleva-Dimitrova et al. 2020). Eleven fatty acids were detected in negative ionization MS/MS spectra. They were determined in *A. tataricus* (Su et al. 2019). The deprotonated molecular ion [M-H]^−^ of peak no. 23 (negative ion mode, Rt = 5.45min) at *m*/*z* 327.2194 with exact molecular formula C_18_H_32_O_5_. It produced a peak at *m*/*z* 229 [M-H-3H_2_O-CO_2_]^−^, referring to the release of three molecules of water indicating the presence of three OH groups in the structure. Releasing 44 amu indicated the decarboxylation of the compound, and by comparing the data with the literature, it was identified as trihydroxyoctadecadienoic acid (Peixoto Araujo et al. 2020). Peaks no. 24, 25, and 27 showed deprotenated molecular ions [M-H]^−^ at *m*/*z* 329.2362, 329.2361, and 329.2361, respectively (negative ion mode, Rt = 5.71, 6.13, 6.77 min, respectively). The mass difference between peak no. 23 and other peaks 24, or 25, or 27 was 2 Da, that indicate the presence of an extra double bond and they were identified as trihydroxyoctadecadienoic acid and trihydroxyoctadecenoic acid, respectively (El-Hawary et al. 2020). The deprotonated molecular ion [M-H]^−^ of peak no. 24 (negative ion mode, Rt = 5.71 min) with exact molecular formula C_18_H_34_O_5_. The fragmentation pattern produced the fragment ion at *m*/*z* 229 [M-H-100]^−^ due to the releasing of C_6_H_12_O moiety, followed by the releasing of water molecule which gives a fragment ion at *m*/*z* 211[M-H-118]^−^, after that cleavage of the C–C single bond attached to OH group at C-9 that was expressed as the fragment ion at *m*/*z* 171, this compound was identified as pinellic acid and these data were matched with the previous reported data (Nadeem et al. 2020). The deprotonated molecular ion [M-H]^−^ of peak no. 28 (negative ion mode, Rt = 7.42 min) at *m*/*z* 313.2406 with exact molecular formula C_18_H_34_O_4_. The releasing of water molecule led to the production of base peak at *m*/*z* 295 [M-H-18]^−^. Indeed, the release of the octane group appeared at *m*/*z* 201[M-H-C_8_H_16_]^−^, these fragmentation patterns matched with literature, and identified as dihydroxyoctadecenoic acid (Zheleva-Dimitrova et al. 2020). The mass difference between peak no 29 and peak 30 was 2 Da, that indicating the presence of extra double bond and they were identified as Hydroxyoctadecatrienoic acid and Hydroxyoctadecadienoic acid, respectively.The deprotonated molecular ion [M-H]^−^ of peak no. 29 (negative ion mode, Rt = 7.91 min) at *m*/*z* 293.2143, with exact molecular formulas C_18_H_30_O_3_. The fragmentation pattern showed the loss of water molecule [M-H-18]^−^ at* m*/*z* 275 which indicates the presence of one allylic OH group in the structure (Peixoto Araujo et al. 2020). The deprotonated molecular ion [M-H]^−^ of peak no. 30 (negative ion mode, Rt = 8.40 min) at *m*/*z* 295.2297 with exact molecular formula C_18_H_32_O_3_. The molecular ion of base peak at *m*/*z* 277 [M-H-18]^−^ appeared due to the loss of water molecule. The cleavage of the double bond between (C12–C11) that led to the appearance of the abundant ion at *m*/*z* 113 [M-H-C_11_H_18_O_2_]^−^. By comparing fragmentation patterns with data in the literature, it was identified as hydroxyoctadecadienoic acid (Nadeem et al. 2020). The deprotonated molecular ion [M-H]^−^ of peak no. 31(negative ion mode, Rt = 9.33 min) at *m*/*z* [−]255.2339 with exact molecular formula C_16_H_32_O_2_. The fragmentation pattern involved fragment ion at *m*/*z* [M-H-C_2_H_3_O_2_]^–^, and *m*/*z* 44 [M-H-C_2_H_3_O_2_-CHO_2_]^−^, which matched with literature and identified as Palmitic acid (Made Ratih et al. 2019). The deprotonated molecular ion [M-H]^−^ of peak no. 32 (negative ion mode, Rt = 9.90 min) at *m*/*z* 271.2281 with exact molecular formula C_16_H_32_O_3_. The fragmentation pattern involved fragment ions at *m*/*z* 225 [M-H-C_15_H_29_O] ^−^, *m*/*z* [M-H-C_15_H_29_O-C_2_H_3_O_2_] ^−^, and *m*/*z* 44[M-H-C_15_H_29_O-C_2_H_3_O_2_-CHO_2_]^–^.That was matched with reported data of hydroxyhexadecanoic acid (Juniperic acid) (Made Ratih et al. 2019; Zhang et al. 2023). The deprotonated molecular ion [M-H]^−^ of peak no. 33 (negative ion mode, Rt = 10.89min) at *m*/*z* 299.2582 with exact molecular formula C_18_H_36_O_3_. Characteristic fragment ion at *m*/*z* 255 [M-H-44]^−^ for the releasing of carboxyl group. By comparing fragmentation patterns with data in literatures, it could be tentatively identified as Hydroxyl stearic acid (C_18_H_36_O_3_) (Made Ratih et al. 2019; Peixoto Araujo et al. 2020).

## Discussion

**Fig. 11:**
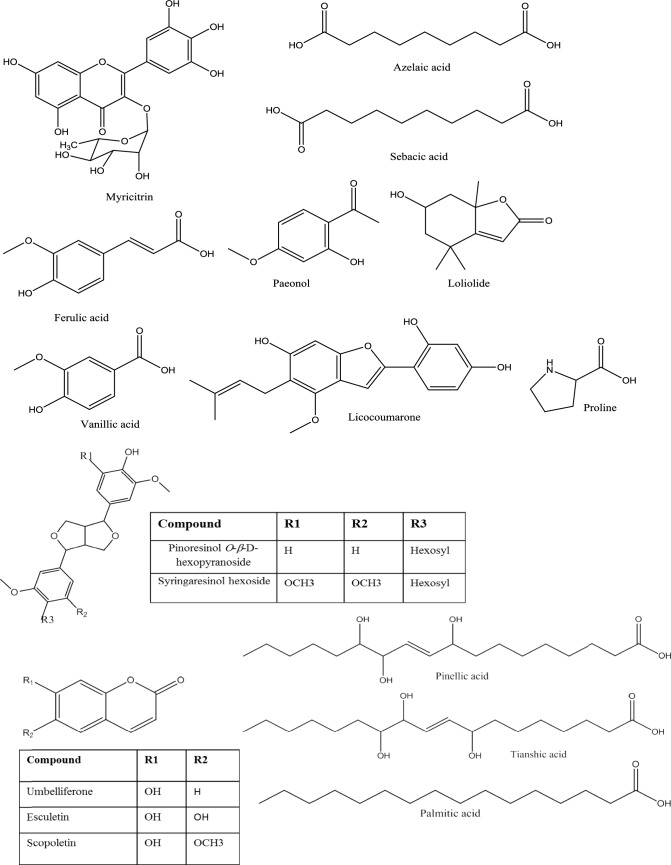
Chemical structures of metabolites identified in methylene chloride fraction of stalk of *A. chinensis*

According to the World Health Organization, psoriasis is a chronic autoimmune inflammatory non-communicable disease that affects people of all ages and has no preference for gender. It can affect the skin, nails, and joints and is linked to several complications (Michalek et al. 2017). The pathophysiology of psoriasis is recognized with a massive release of pro-inflammatory cytokines and chemokines that lead to epidermal hyper proliferation, skin erythema, and scaly skin plaques (Liu et al. 2007). The immunosuppressive drugs such as methotrexate and cyclosporine were considered as the conventional treatments for psoriasis (Koo 1999). However, may cause serious adverse effects (El-Shemy 2017). Definitely, systemic treatment with methotrexate and cyclosporine damages liver and kidney functioning and decreases RBCs, WBCs, and Platelets counts (Agrawal et al. 2010). Additionally, their topical application is associated with poor permeation through the skin because of its hydro-solubility and greater molecular weight (Chen et al. 2015a; Pinto et al. 2014). Therefore, finding a natural plant resource with therapeutic effects against psoriasis is a global demand. In previous studies, the antioxidant activity of the ethyl acetate and *n*-butanolic leaves extracts of *A.squamatus* (Boulechfar et al. 2014) and the ethanolic flower extract of *A.chinensis* (Kakodkar et al. 2019)*,* a natural medicinal resource, have been confirmed, and anti-inflammatory activity of other species of *Aster* (Su et al. 2019; Ngabire et al. 2018; Choi et al. 2017). Hence, the current study investigated for the first time the anti-psoriatic activity of *A.squamatus* herb, as well as stalk and flowers of* A.chinensis* extracts and Bio-guided fractionation of *A. chinensis* stalk alcoholic extract against IMQ-induced psoriasis. A further study was performed on the most active fraction, methylene chloride from *A. chinensis* stalk to focus on elucidating the bioactive components that may be responsible for the observed effects via the UHPLC-MS/MS technique (Fig. 11). Moreover, the aim was extended to delineate the possible mechanisms involved. The IMQ-treated mouse model is considered one of the most broadly used models to study psoriasis (Chamcheu et al. 2016). The reason is that it faithfully resembles human plaque-type psoriasis concerning skin erythema, thickening, scaling, as well as to inflammatory infiltrate (van der Fits et al. 2009). IMQ results in inducing an immune response in the body by resembling as an agonist of (TLR) 7 and 8 (Zhou et al. 2021). This stimulation prompts lymphocyte activity that may lead to splenomegaly. Additionally, IMQ stimulates the release of inflammatory factors such as IL-1β, IL-6, IL-23, and IL-17 (Su et al. 2022) leading to produce psoriasis-like symptoms in the skin, including erythema, scaling, and epidermal thickening. Additionally, the significant increase in MDA content and the reduction in SOD activity in IMQ-treated mice indicate heightened oxidative stress (Chen et al. 2017). This oxidative stress promotes the release of damage-associated molecular patterns (DAMPs) such as high mobility group box-1 (HMGB-1). HMGB-1, in turn, activates TLR4, which triggers NFκBp65 activation (Wu and Yang 2018; Mohamed et al. 2023). This activation results in the production of pro-inflammatory cytokines, including TNF-α, IL-1β, and IL-6, thereby perpetuating a positive feedback loop of inflammation (Su et al. 2022; Khedr et al. 2024). Notably, IL-1β binds to its receptor, initiating the IL-IR signaling cascade, which is crucial for the early differentiation of Th17 cells. Th17 cells exacerbate neuroinflammation and are associated with the massive production of Il-23 and IL-17 in psoriatic patients (Lowes et al. 2014).

The data presented in this study expressed comparing and bio-guided for the first time the anti-psoriatic activities for the topical application of the three plant extracts of *A. squamatus*, *A. chinensis* flower, and stalk separately (100 mg/kg). Subsequently, various fractions of *A. chinessis* stalk (the most potent extract) were created to determine which fraction contributed to its biological activity. These fractions, including petroleum ether, methylene chloride, ethyl acetate, and butanol, were applied topically and tested separately. Based on the evaluation of PASI scores and histopathological examination, the methylene chloride fraction of *A. chinensis* stalk exhibited the highest protective efficacy against IMQ-induced psoriasis-like pathological features. Throughout the study, all groups were monitored for signs of erythema, scaling, and skin thickness. The biochemical mechanisms behind the anti-psoriasis effects of the methylene chloride fraction of *A. chinensis* stalk could be attributed to a decrease in oxidative stress, evidenced by increased SOD activity and reduced MDA content. This reduction in oxidative stress could lead to decreased release of HMGB-1, thereby inhibiting TLR-4 activation. Consequently, this suppresses NFKB p65 activation, leading to a decrease in serum levels of IL-1β, IL-6, IL-23, and IL-17. Furthermore, improvements were noted in dorsal skin erythema, scaling reduction, and decreased epidermal thickness, as reflected in the PASI score. Additionally, there was a noticeable decrease in dermal inflammatory cell infiltration. Indeed, the effect of topical treatment of methylene chloride fraction of *A. chinensis* stalk is comparable to the standard treatment (mometasone). The observed effectiveness of the methylene chloride fraction from *A. chinensis* stalk against psoriasis could be attributed to its polyphenol compounds. These compounds, which contain one or more hydroxyl groups in their structure, and classified in to flavonoids and non-flavonoids classes (García-Pérez et al. 2017). Some phenolic compounds can also inhibit pro-inflammatory mediator's activity or gene expression, such as cyclooxygenase (COX). Phenolic compounds can also up- or down regulate transcriptional elements involved in antioxidant pathways, such as nuclear factor-κB (NF-κB), these compounds believed to reduce oxidative stress either individually or in combination (Contardi et al. 2021).This reduction, in turn, suppresses inflammatory pathways and suggests a potential therapeutic application for treating psoriasis (Xie et al. 2021). Flavonoids are widely distributed in plants and considered as secondary metabolites (García-Perez et al. 2017). Flavonoids have been exposed a wide range of medicinal properties. It possesses a significant role in treatment of psoriasis due to their anti-inflammatory effect (Xie et al. 2021). Hydroxyluteolin was isolated from *A.chinensis* and reported in literature (Bhargav et al. 2018), could be decrease apoptosis and inhibit tumor growth via suppression DNA alteration, additionally the anti-oxidant and anti-inflammatory effects due to ROS scavenger, inhibiting IL-6, iNOS, and pro-inflammatory enzymes LOX and COX-2 (López-Lázaro 2009). The predominant flavonoid of the flower extract of *A.chinensis* was myricetin and myricetin-3-Orhamnoside (Bhargav et al. 2018). Additionally, in traditional medicine, the leaves extract of *Syzygium formosum* plant, belongs to Myrtaceae family, have been used as treatment for psoriasis and other dermatitis, that contain 54% of myricetin-3-*O*-rhamnoside of total flavonoids. Moreover, the anti-inflammatory mechanism of myricetin-3-*O*-rhamnoside was reducing COX-2, IL-1β, IL-6, and IL-8 (Hoang et al. 2021). On the other hand, the poly phenolic acids are a compound has one or more hydroxyl groups involved to aromatic ring, that have broad range of medicinal uses (García-Perez et al. 2017). In the previous years, hydroxycinnamic acids and derivatives have been considered and advantageous in several research fields for their anti-inflammatory, skin diseases, and anti-psoriasis activities (Contardi et al. 2021). One of the most common of hydroxycinnamic acid is ferulic acid and isoferulic acid that used as anti-inflammatory drug in Japanese Oriental medicines, by decreasing IL-8 and cyclooxygenase and nitric oxide synthase resulting to decrease pro-inflammatory cytokines and mediators (Batista 2014). Also, vanillic acid that isolated from *A. squamatus* herb extract (Sperotto et al. 2002) has anti-inflammatory effect by decreasing the oxidative stress, stop activation of NFκB, suppressed neutrophils production and cytokines. That is why it can be used as analgesic and anti-inflammatory drug (Calixto-Campos et al. 2015). Caffeic acid derivatives that found in the aqueous extract of *A. squamatus* herb (Sperotto et al. 2002), were used in cosmetics preparation, have the ability to decrease oxidative stress and inflammation specially occur on the skin, by suppressing the neutrophil elastase, IL-6, and TNF-α (Silva et al. 2014). In China, the ancient Chinese people made a medicinal herbal formula used for treatment of psoriasis, and the main constituent is Caffeoylquinic acid (Chen et al. 2015b). On the other studies, the chlorogenic acid and its derivatives have the ability to suppress the pro-inflammatory cytokines and decrease the inflammatory symptoms (Elkhawaga et al. 2023). Many studies on salicylic acid, and its isomers of hydroxyl benzoic acid, proved its important role of psoriasis treatment. That is why, the ability of salicylic acid to increase the permeation of the momentasone furoate drug through the skin and decrease the other side effect (Xie et al. 2021). The latest study about vanillic acid, protocatechuic acid (3,4-Dihydroxybenzoic acid), and gentisic acid (2,5-Dihydroxybenzoic acid)showed their abilities to suppress IL-23 expression and PASI score in IMQ-induced psoriasis mouse model (Wang et al. 2024). Another chemical compounds found in plants in wide range and have known anti-inflammatory activity, promising candidates for the development of new drugs, could decrease the pro-inflammatory cytokines and chemokines expression are coumarines (Xie et al. 2021). Umbellifrone that isolated from flower petals extract of *A.chinensis* (Bhargav et al. 2018), inhibit the inflammation by different pathways, by suppression the inflammatory cytokines and chemokines, decreasing the thickness of lesion, weight and size of spleen, inhibiting the serum level of IL-4, TNF-α, and mast cells infiltration (Lim et al. 2019). Additionally, Loliolide, a monoterpenoid hydroxylactone, has anti-inflammatory, wound healing, and anti-apoptosis effect for the damaged skin via decreasing reactive oxygen species (ROS) and enhancing the countenance of the impairment repair-related gene SIRT1 (Park et al. 2019). Also, Esculetin, dihydroxy coumarin, showed in different studies on Imiquimod (IMQ)-induced psoriasis in mouse models, has a magical power to suppress NF-κB activation, PASI scores, and improved skin immunopathology (Chen et al. 2018). It has the ability to suppress IL-6making in the TNF-α, indicating its anti-inflammatory effect (Gao 2010). On the other study showed that coumarin and scopoletin isolated from other plant such as fennel fruit have anti-inflammatory effect by inhibiting IL-6, TNF-α in LPS-induced RAW 264.7 cells (Yang et al. 2015b). In previous study, the unsaturated trihydroxy fatty acid such as pinellic acid was responsible for its anti-inflammatory properties by effectively suppressing the lipopolysaccharide (LPS)-induced production of various inflammatory mediators (Shin et al. 2015). Finally, proline has the ability to increase epithelial proliferation, transdermal permeation enhancer, and anti-inflammatory effect by stimulate the detoxification of oxygen species which produced during the inflammation of tissue (Carregaro et al. 2013).

The evaluation of the chemical characteristics of methylene chloride fraction of *A. chinensis* stalks for further clarifying the material basis ensuring the medicinal effect. In this context, we can summarize that methylene chloride fraction of *A. chinensis* stalk is the most effective fractions after comparing and bio-guided study of *A.squamatus*, *A.chinensis* flower, and stalk extracts and fractions. Therefore, it would be interesting to verify whether the activity observed in the present study is due to these compounds or not by further future studies.

## Conclusion

In conclusion, our findings supported methylene chloride fraction of *A. chinensis* stalk, as a novel natural plant resource, in the treatment of psoriasis as it attenuated both the immune and inflammatory responses. It applied its anti-psoriatic effects by suppressing inflammation, evidenced by decreased TLR-4, levels of HMGB1 and NFκBp65 protein contents, serum levels of pro-inflammatory cytokines interleukin (IL)-1β, IL-6, IL-23, and IL-17, increased (SOD) activity, and reduced (MDA) content. Further studies are recommended to evaluate the biological activity of the major identified compounds, either individually as isolated ones or in combinations.

## Supplementary Information

Below is the link to the electronic supplementary material.

Supplementary file1 (PDF 269 KB)

## Data Availability

All data sets obtained and analyzed during the current study are available in the manuscript. Further inquiries can be directed to the corresponding author.

## Abbreviations

MCF: Methylene chloride fraction
MeOH: Methanol
ANOVA: One-way analysis of variance
ELISA: Enzyme-linked Immunosorbent Assay
IL: Interleukin
IMQ: Imiquimod
LOX: Lipoxygenase
LPS: Lipopolysaccharide
PASI: Psoriasis Area and Severity Index
UHPLC-MS/MS: Ultra-high performance liquid chromatography-tandem mass spectrometry
TNF-α: Tumor Necrosis Factor-Alpha
NFκB: Nuclear factor kappa B
HMGB1: High-mobility group B 1
iNOS: Inducible nitric oxide synthase
COX-2: Cyclooxygenase-2
ROS: Reactive oxygen species
TLR4: Toll-like receptor 4
