# Supplementary material for: Efficacy of Aster chinensis aerial parts metabolites in BALB/c mice model of Imiquimod-induced psoriasis skin inflammation
Source: Inflammopharmacology. 2025 Mar 12;33(4):1973–96. doi: 10.1007/s10787-025-01652-x (PMC11991947; doi:10.1007/s10787-025-01652-x)
Supplement: Supplementary file 1 — Supplementary file1 (PDF 269 KB) [file 10787_2025_1652_MOESM1_ESM.pdf]

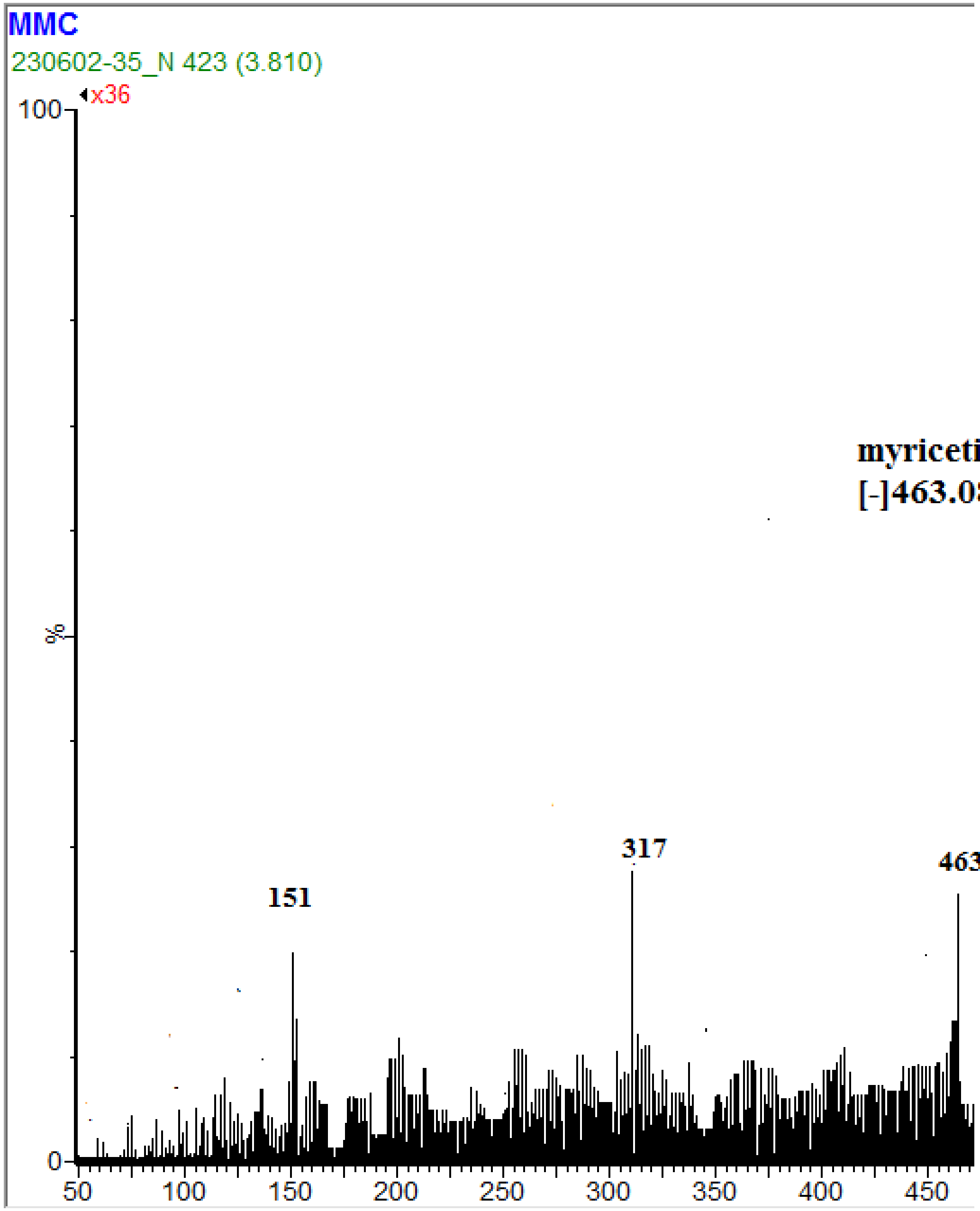

myricetin-rhamnoside
[-]463.0887

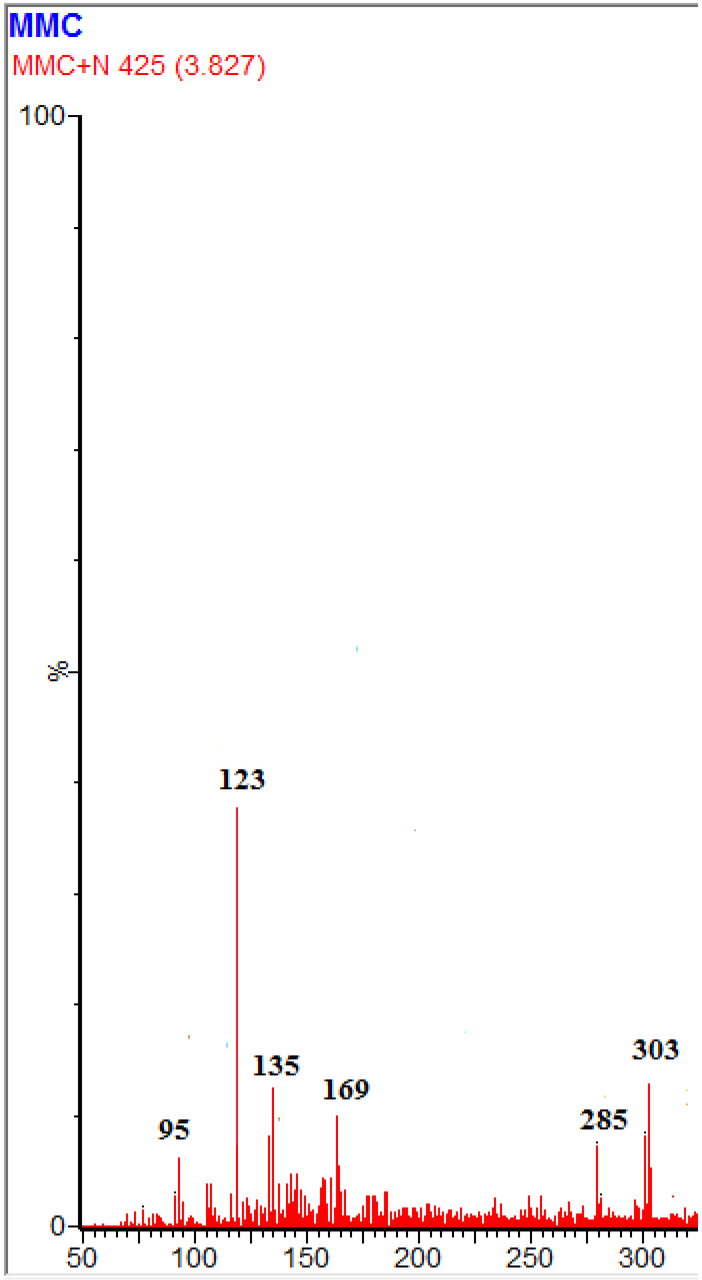

6-Hydroxyluteolin
[+]303.0517

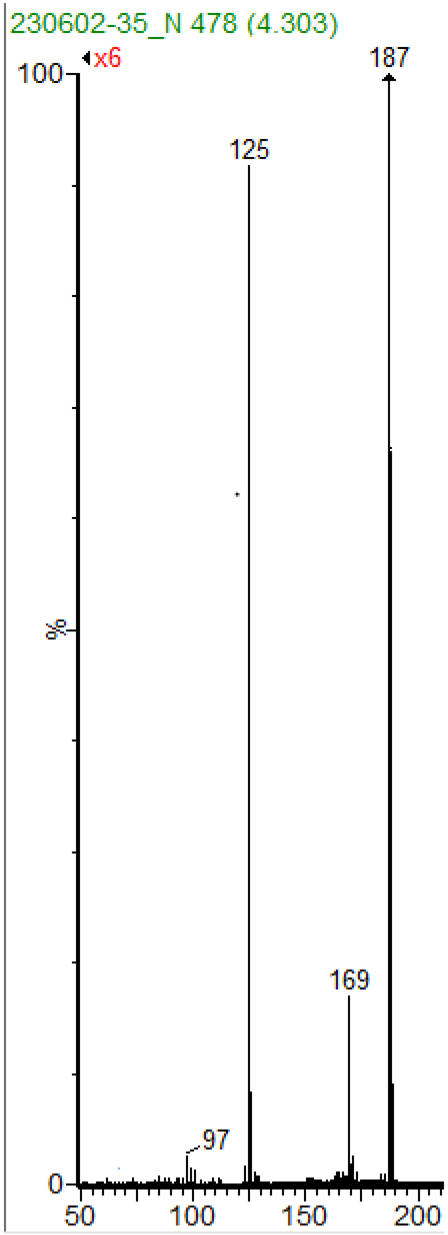

Nonanedioic acid (Azelaic acid)
[-]187.0975

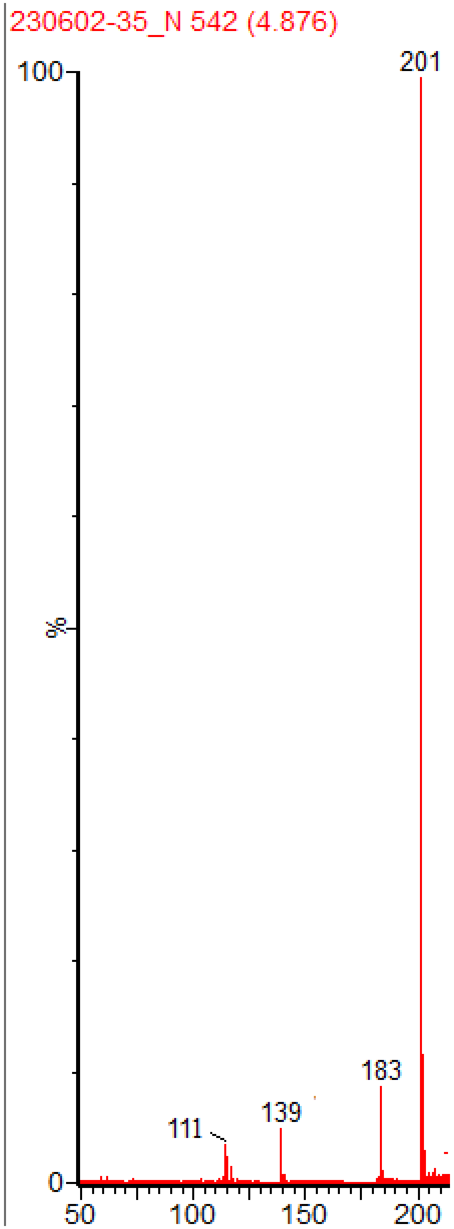

Sebacic acid
[-]201.1134

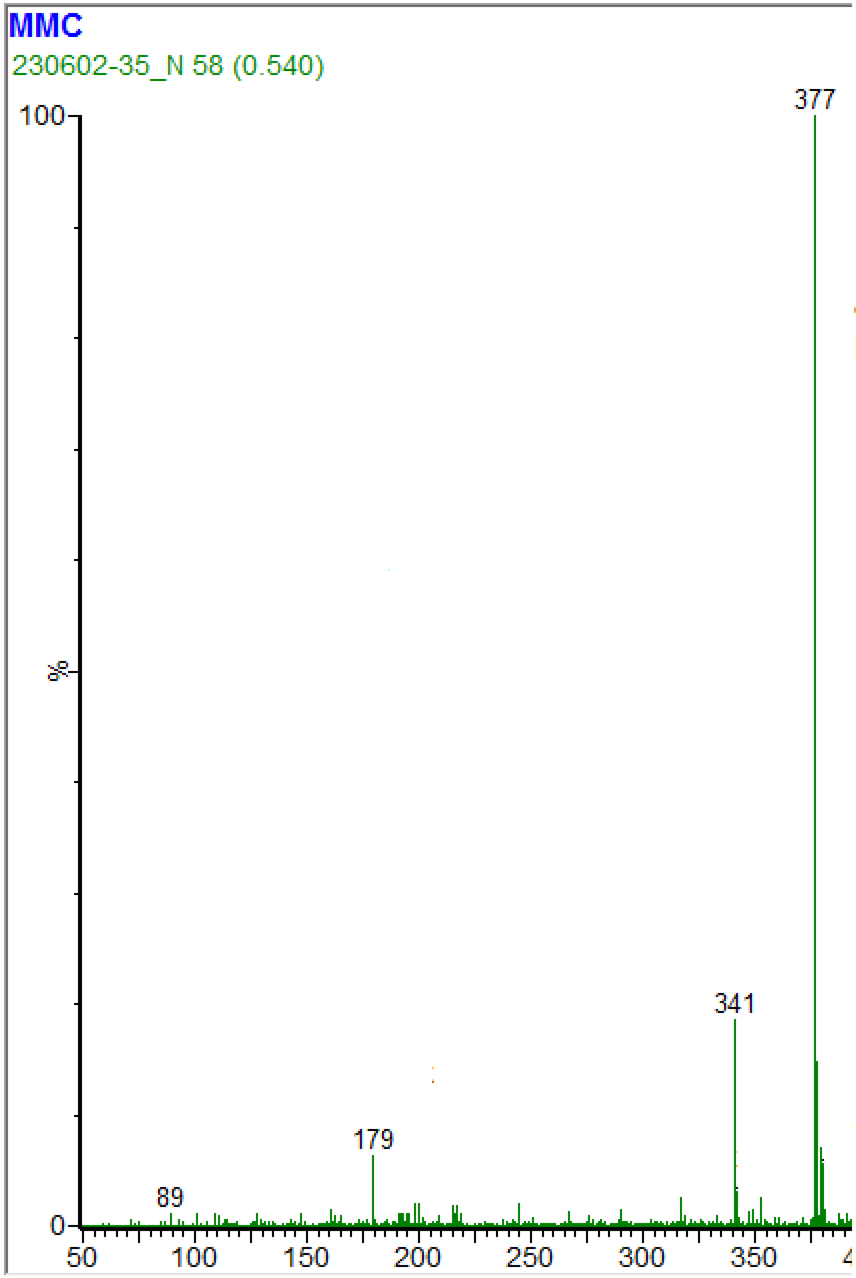

caffeoyl-*O*-hexoside
[-]377.0858

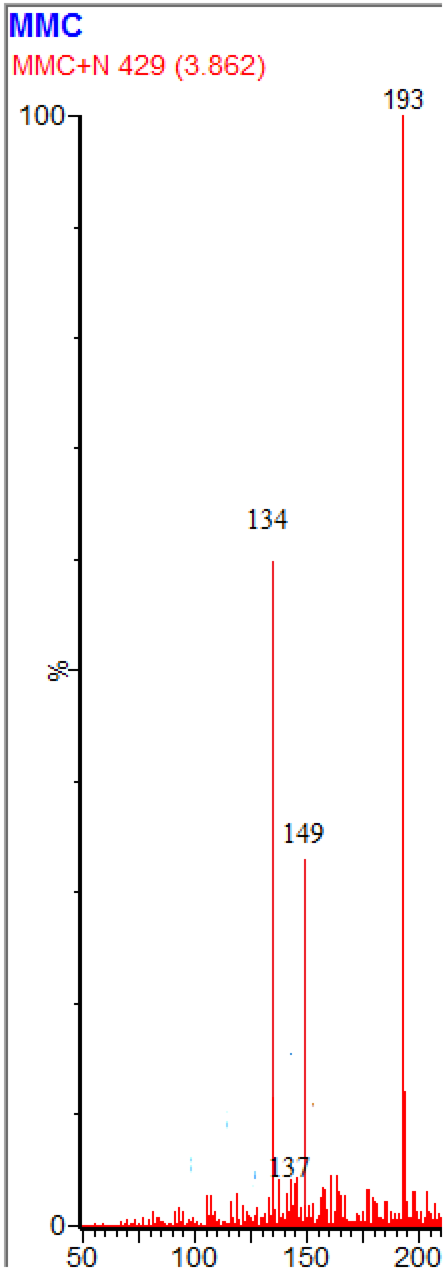

Ferulic acid
[-]193.0509

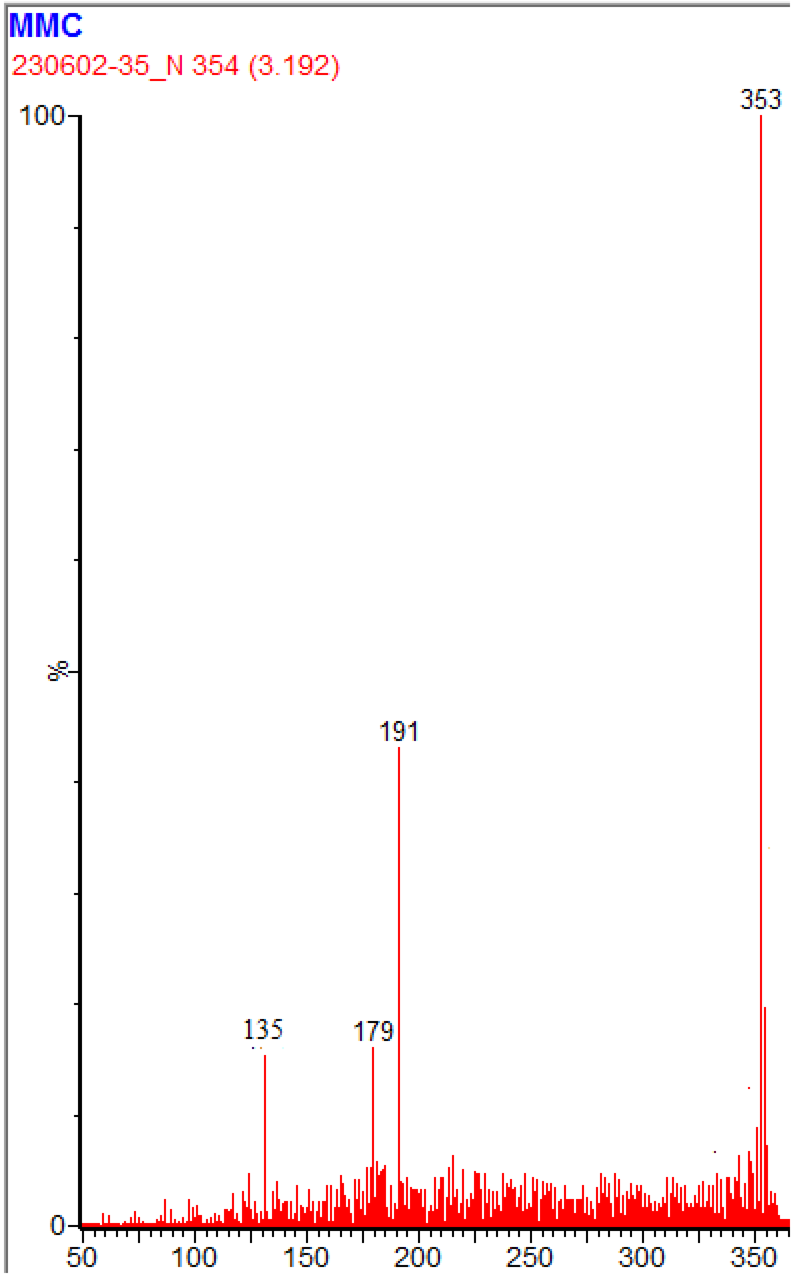

caffeoylquinic acid
[-]353.0872.

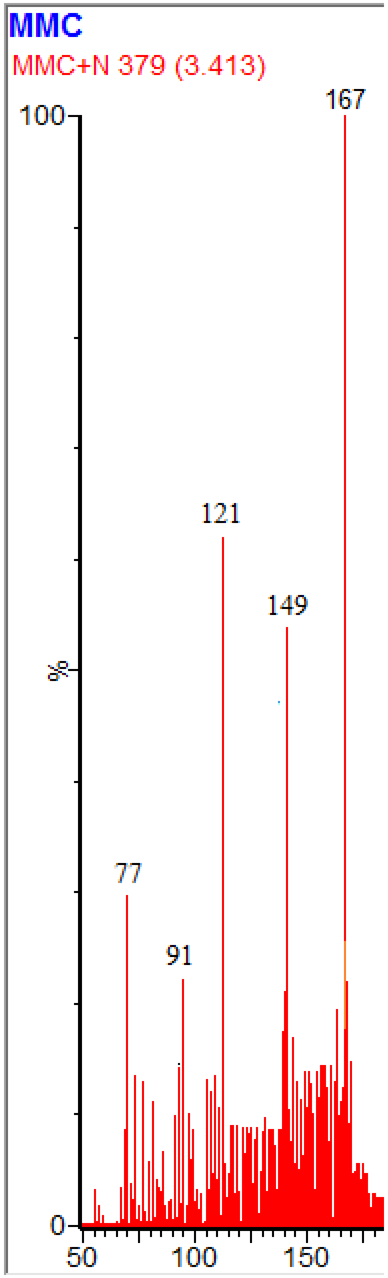

paeonol
[+]167.0713

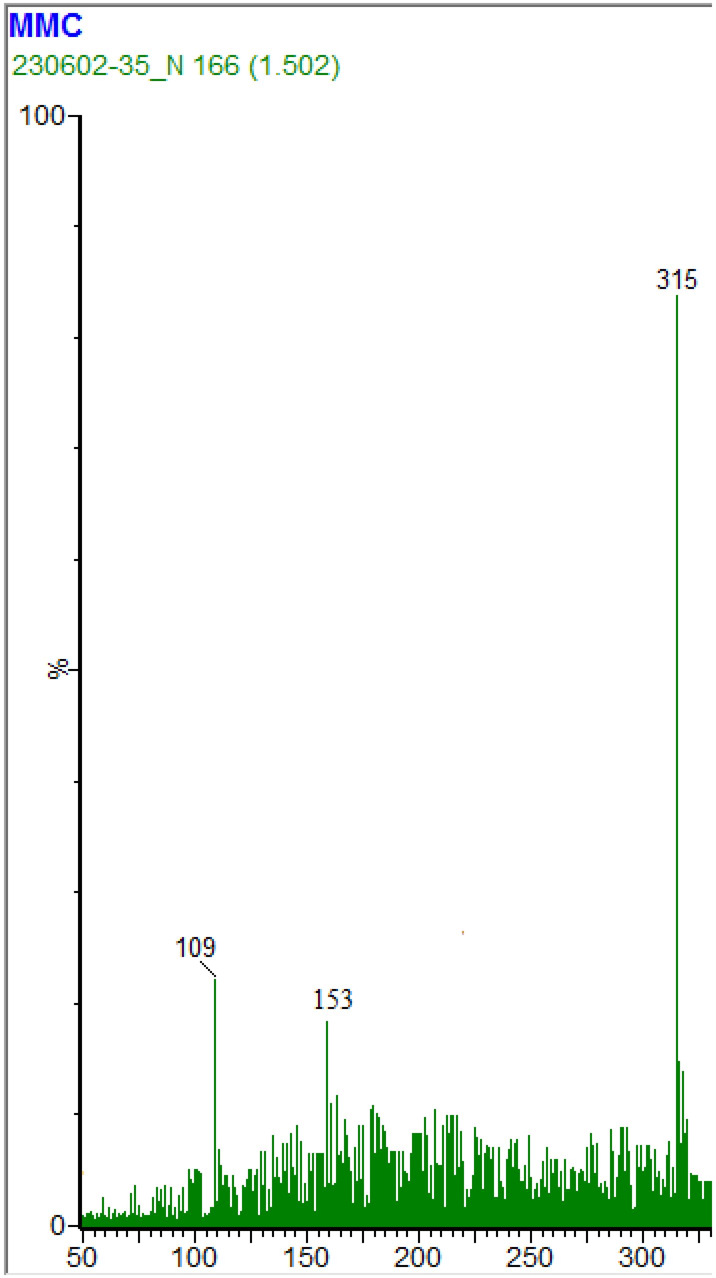

Hydroxyhexosylbenzoic acid
[-]315.0719

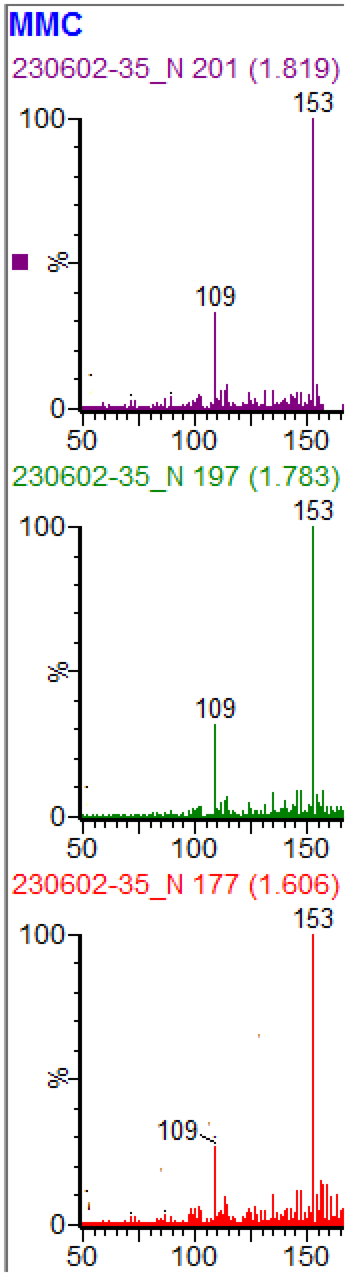

Dihydroxybenzoic acid III

Dihydroxybenzoic acid II

Dihydroxybenzoic acid I

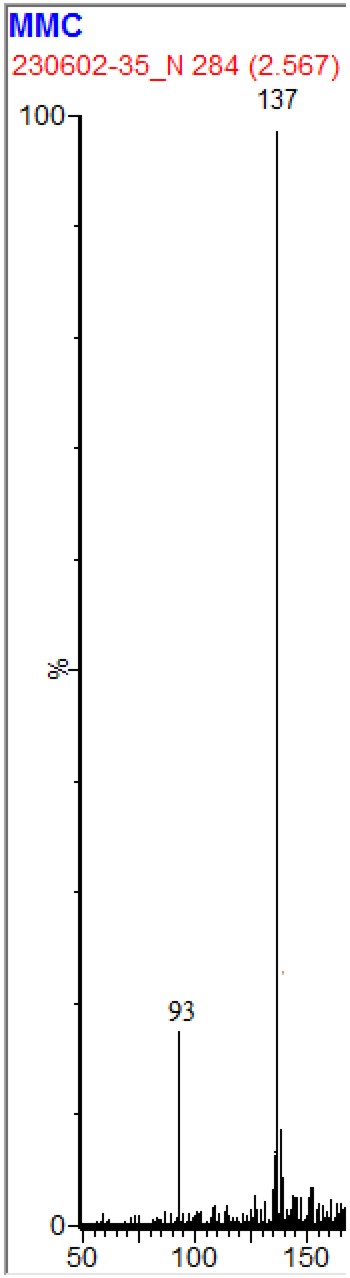

Hydroxy benzoic acid
[-]137.0236

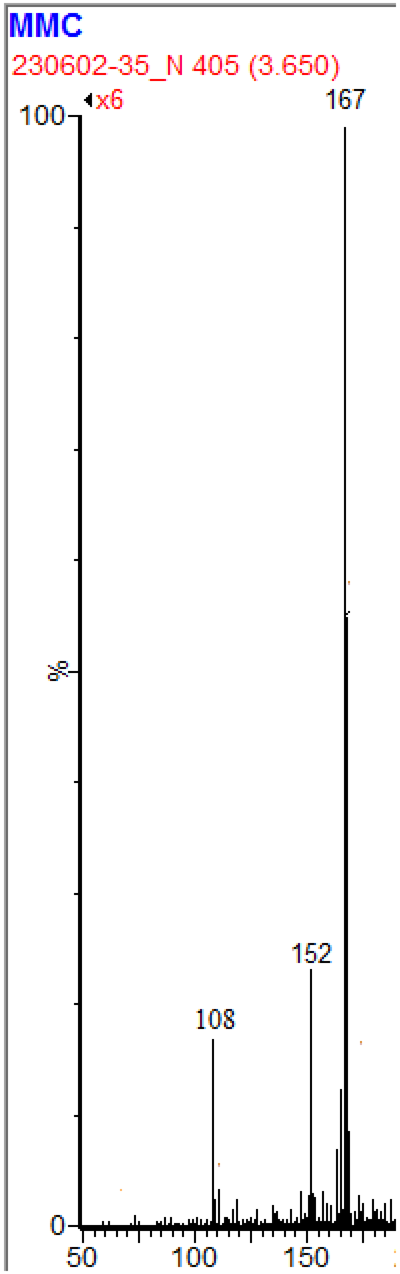

Vanillic acid
[-]167.0348

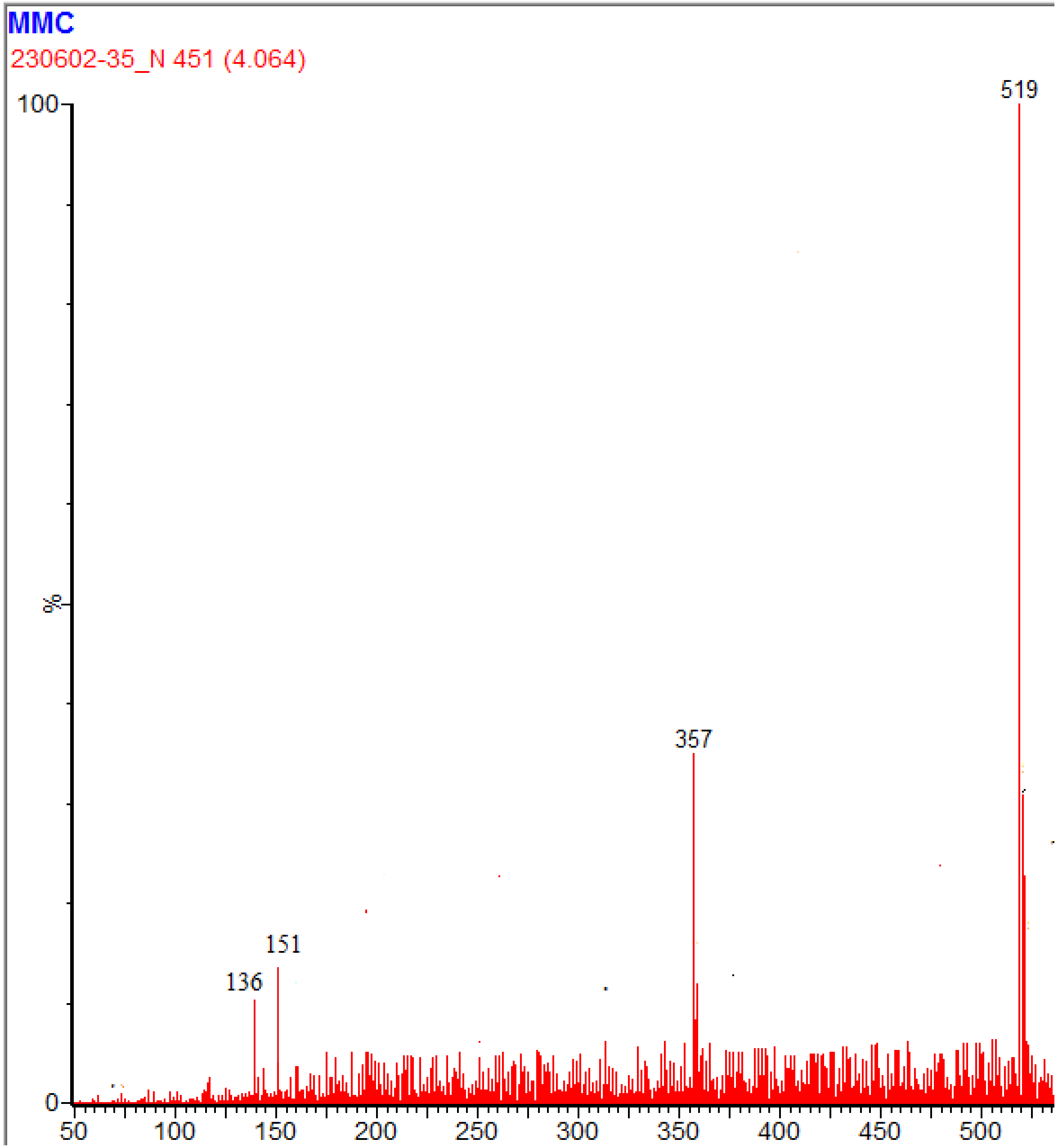

Pinoresinol *O*-*β*-D-hexopyranoside
[-]519.1877

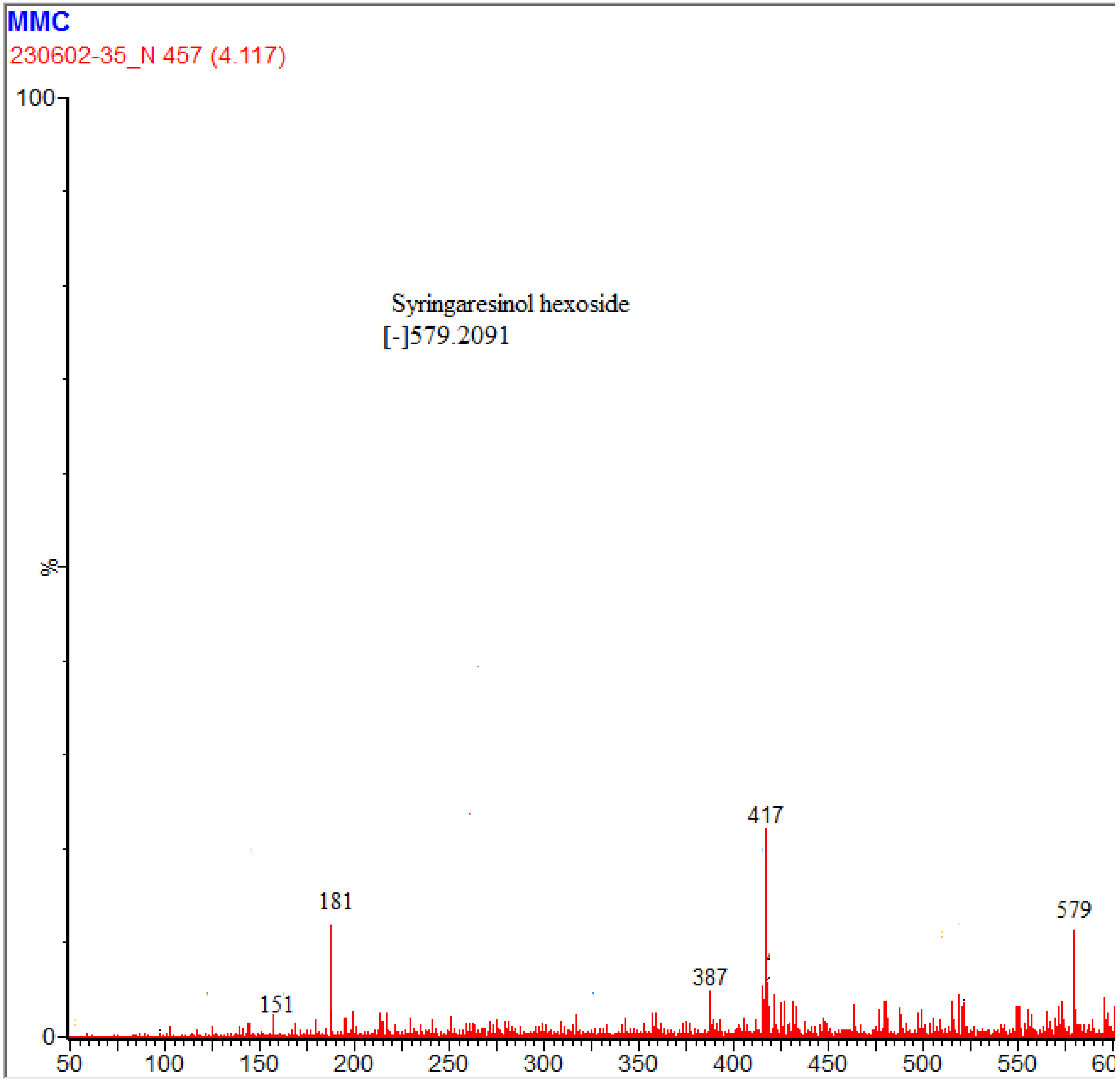

MMC
230602-35_N 457 (4.117)
Syringaresinol hexoside
[-]579.2091
100
%
0
417
181
579
387
151
50
100
150
200
250
300
350
400
450
500
550
60

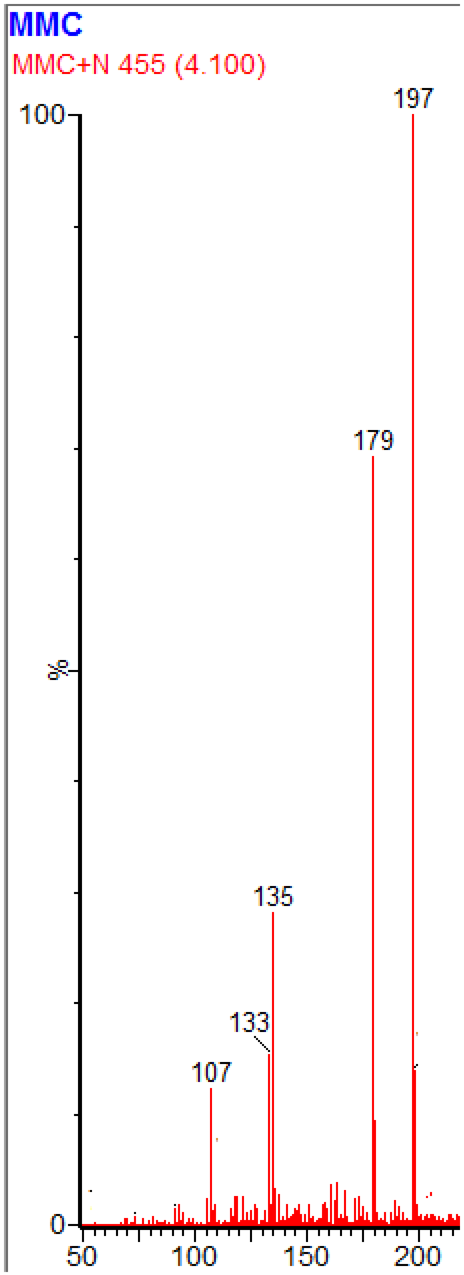

[+]197.1186
Loliolide

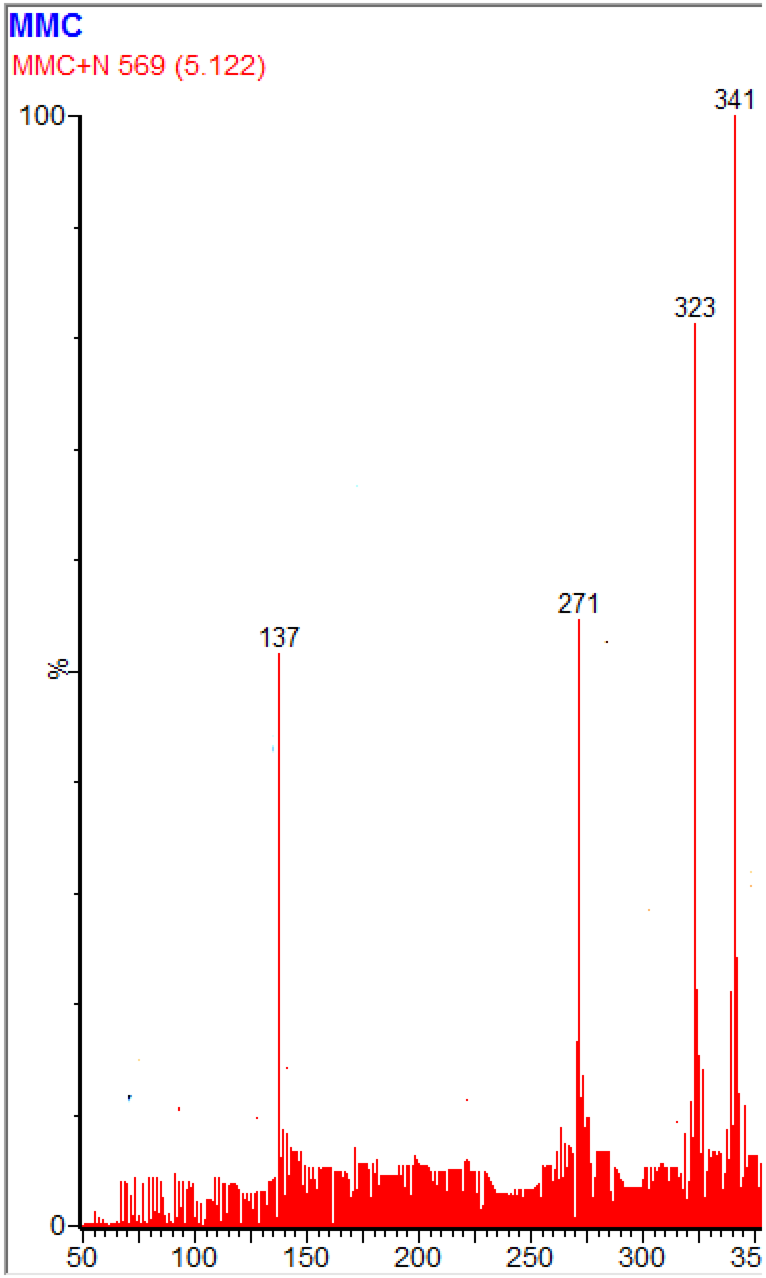

Licocoumarone
[+]341.1412

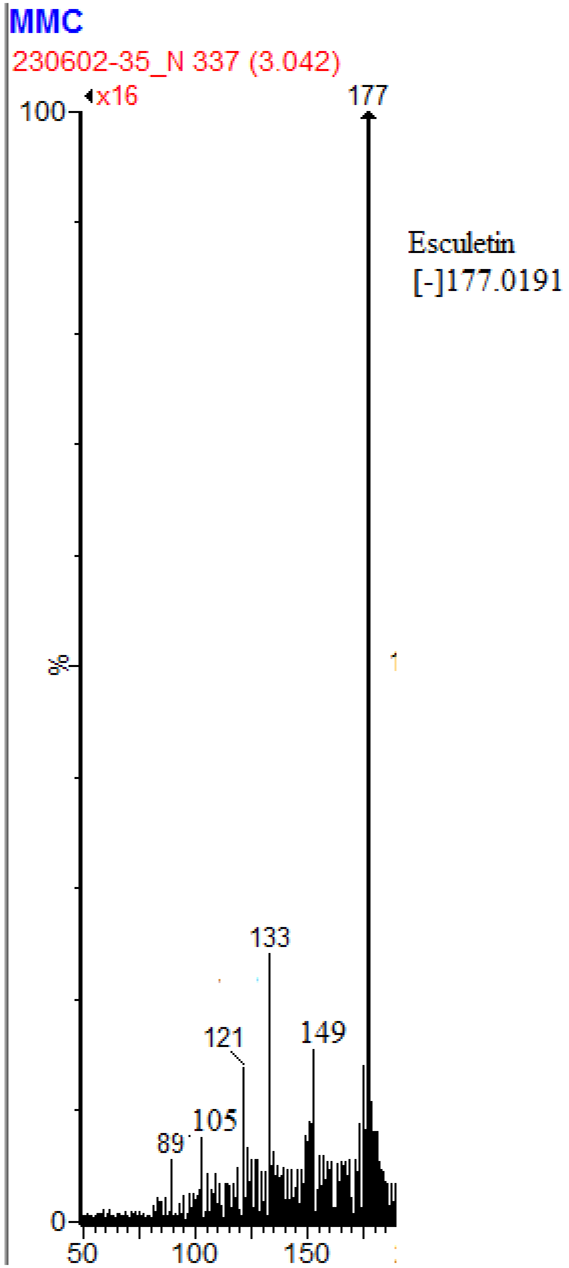

MMC
230602-35_N 337 (3.042)
x16
177
Esculetin
[-]177.0191
100
%
0
133
121
149
105
89
50
100
150

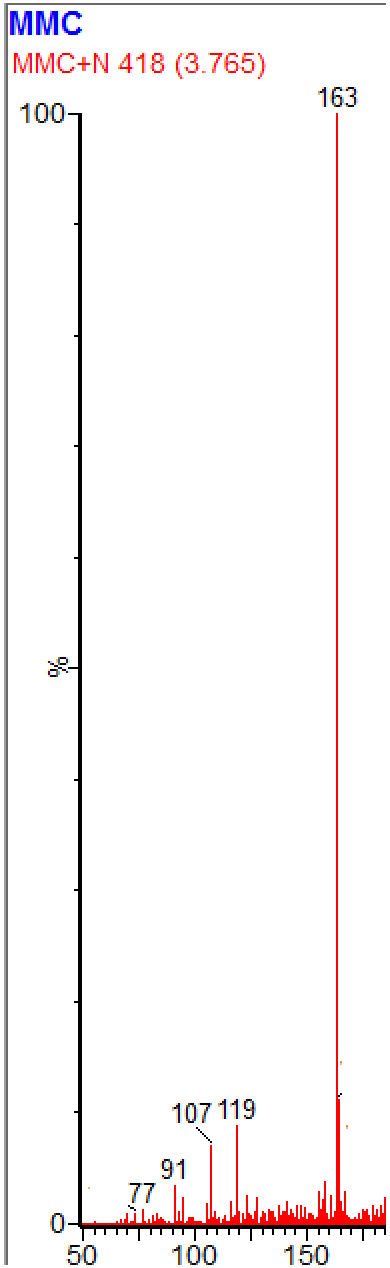

Hydroxycoumarin (Umbelliferone)

[+]163.0402

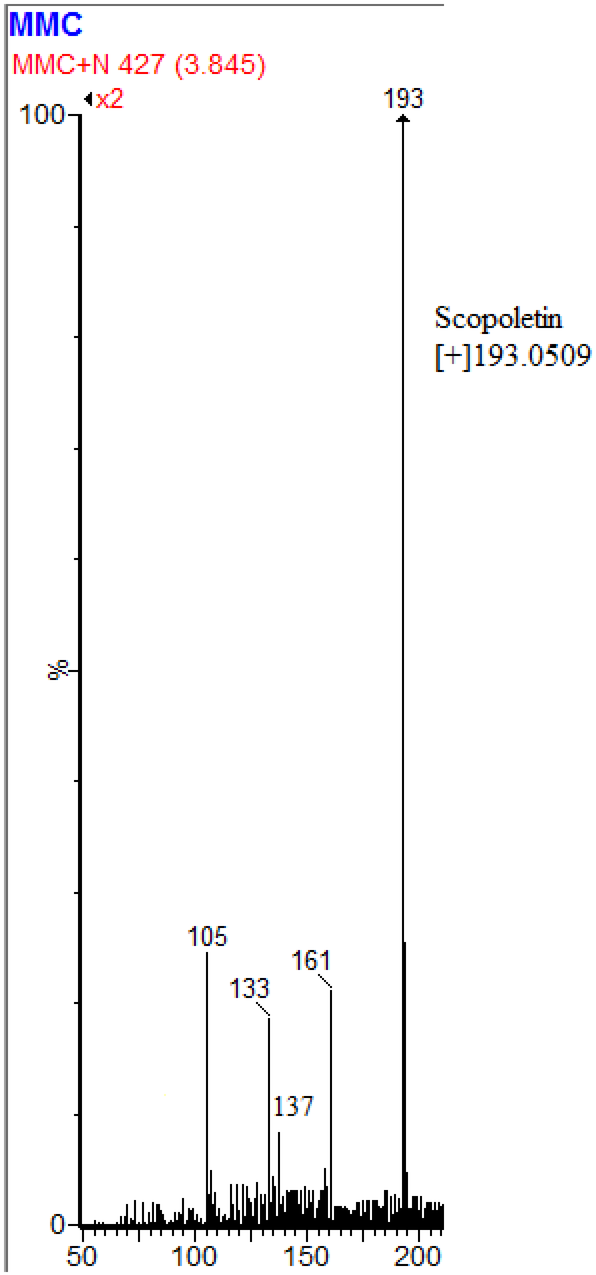
MMC
MMC+N 427 (3.845)
x2
100
%
0
193
105
133
161
137
50
100
150
200
Scopoletin
[+]193.0509

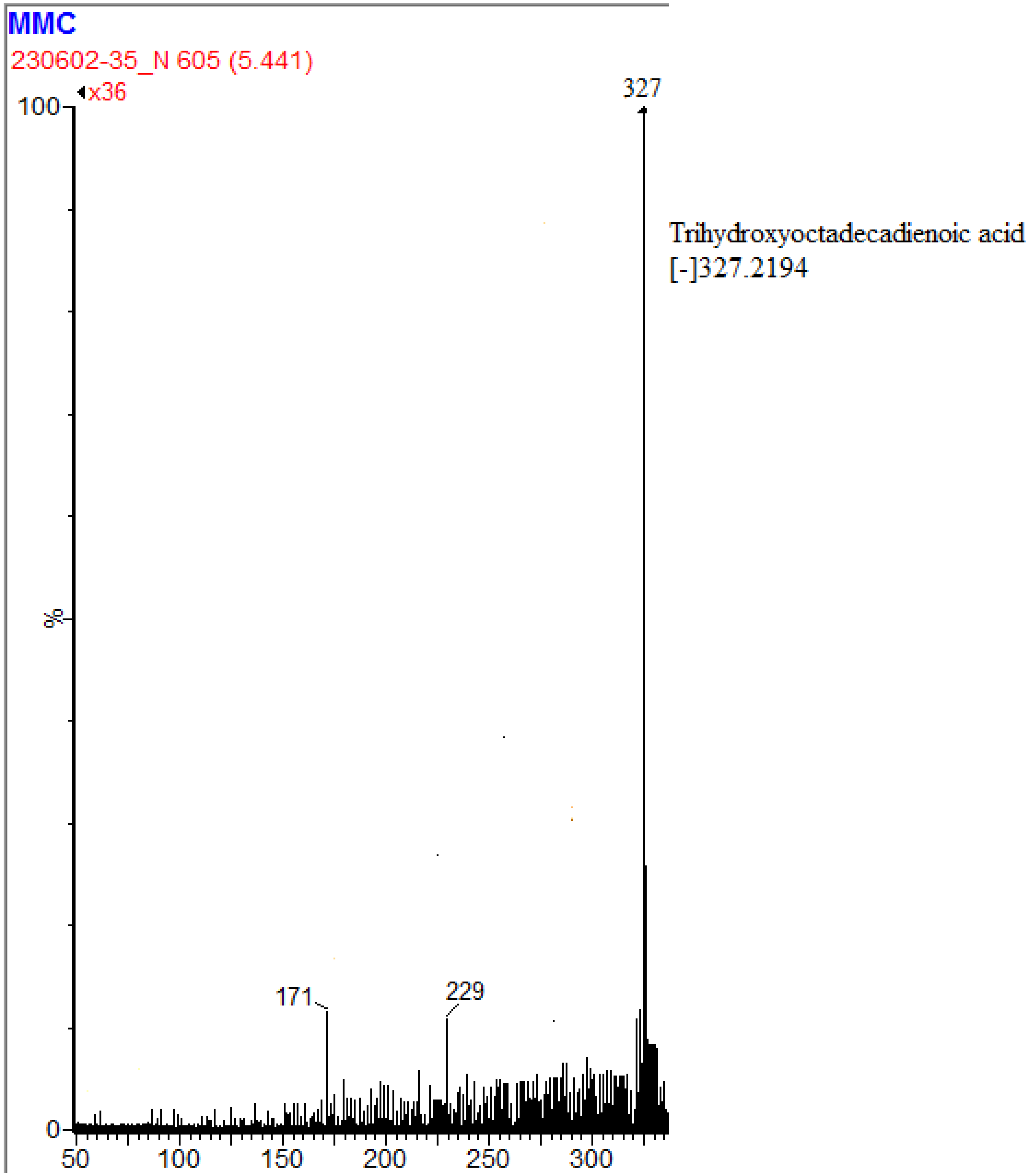

Trihydroxyoctadecadienoic acid
[-]327.2194

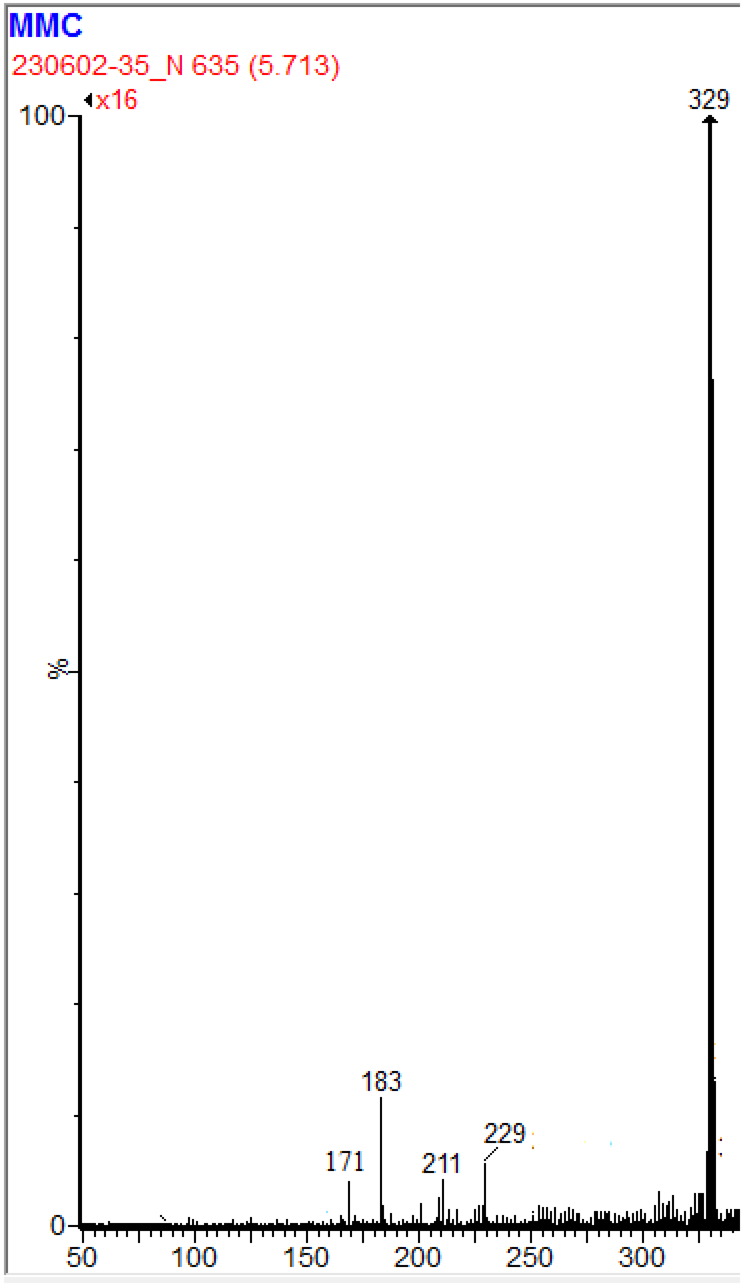

Pinellic acid
[-]329.2362

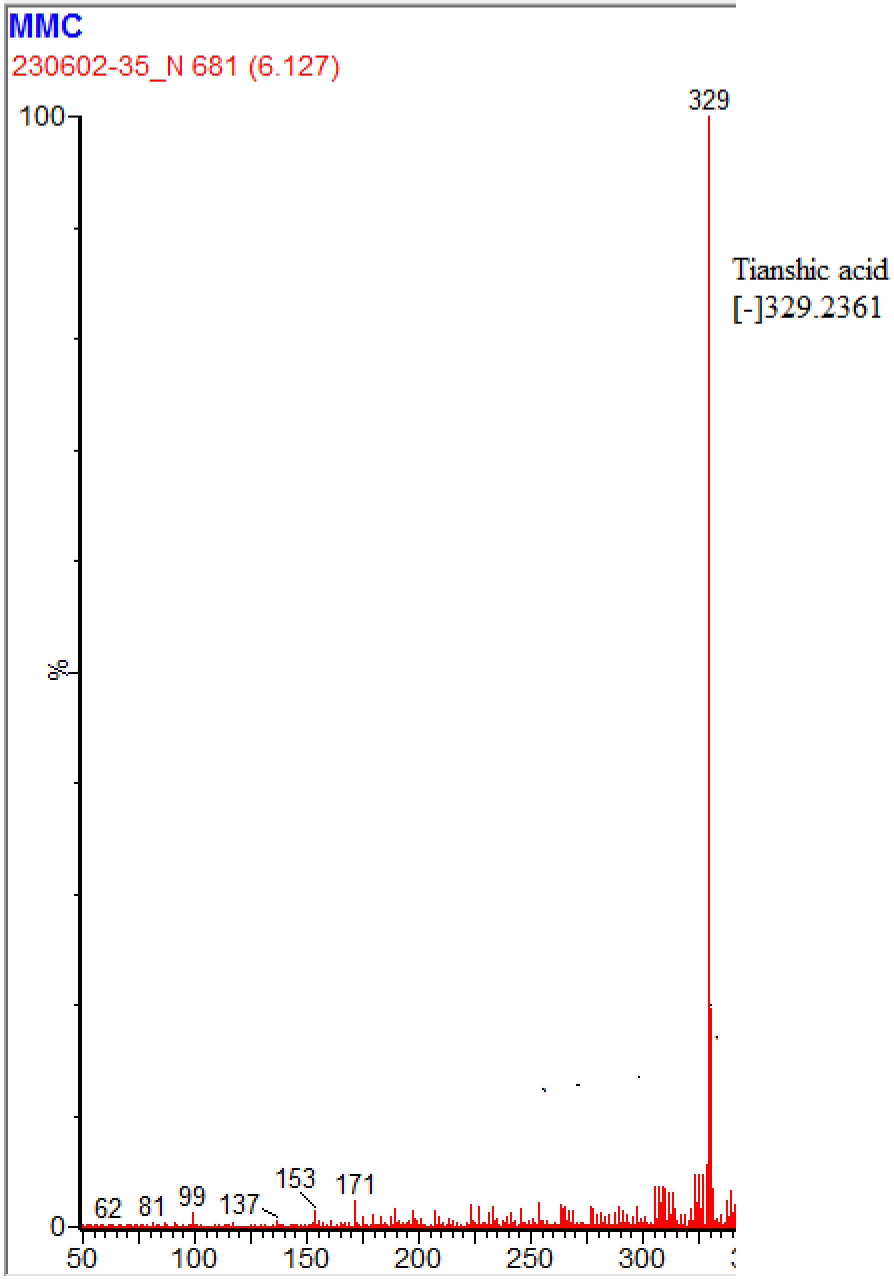
MMC
230602-35_N 681 (6.127)
329
100
%
0
62
81
99
137
153
171
50
100
150
200
250
300
Tianshic acid
[-]329.2361

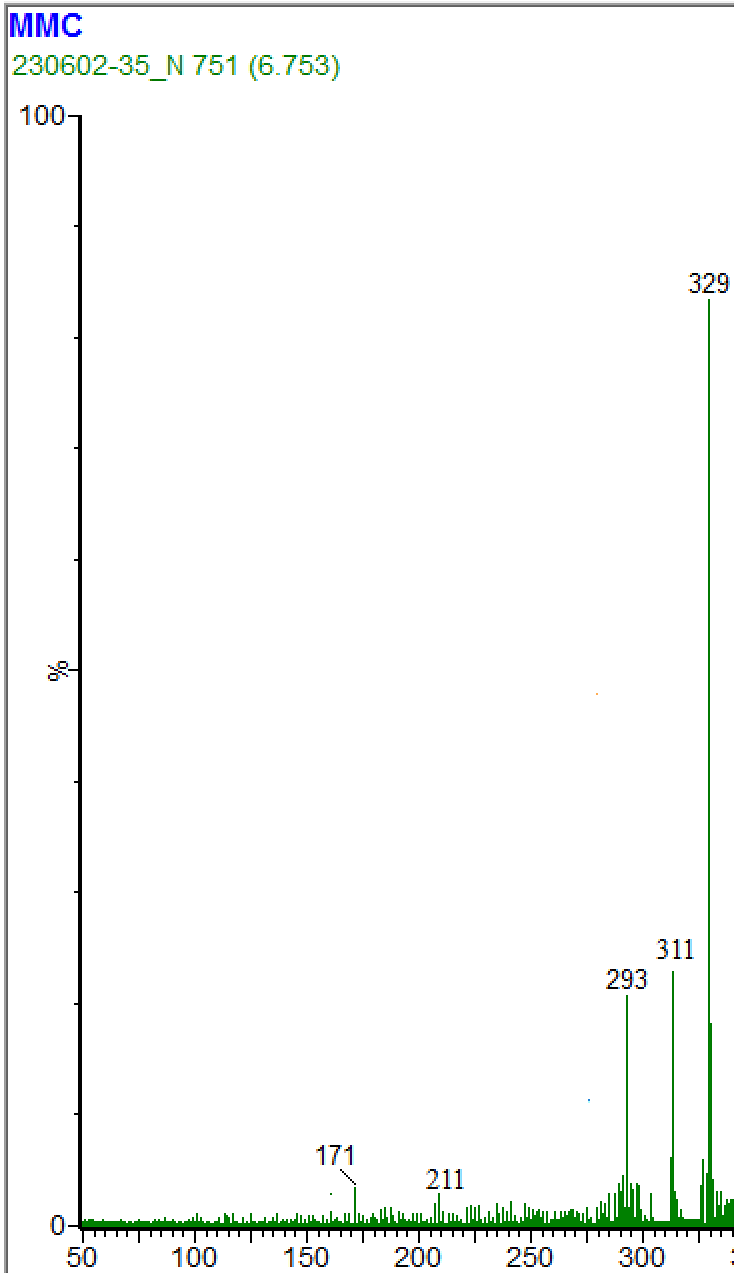

Trihydroxyoctadecenoic acid
[-]329.2361

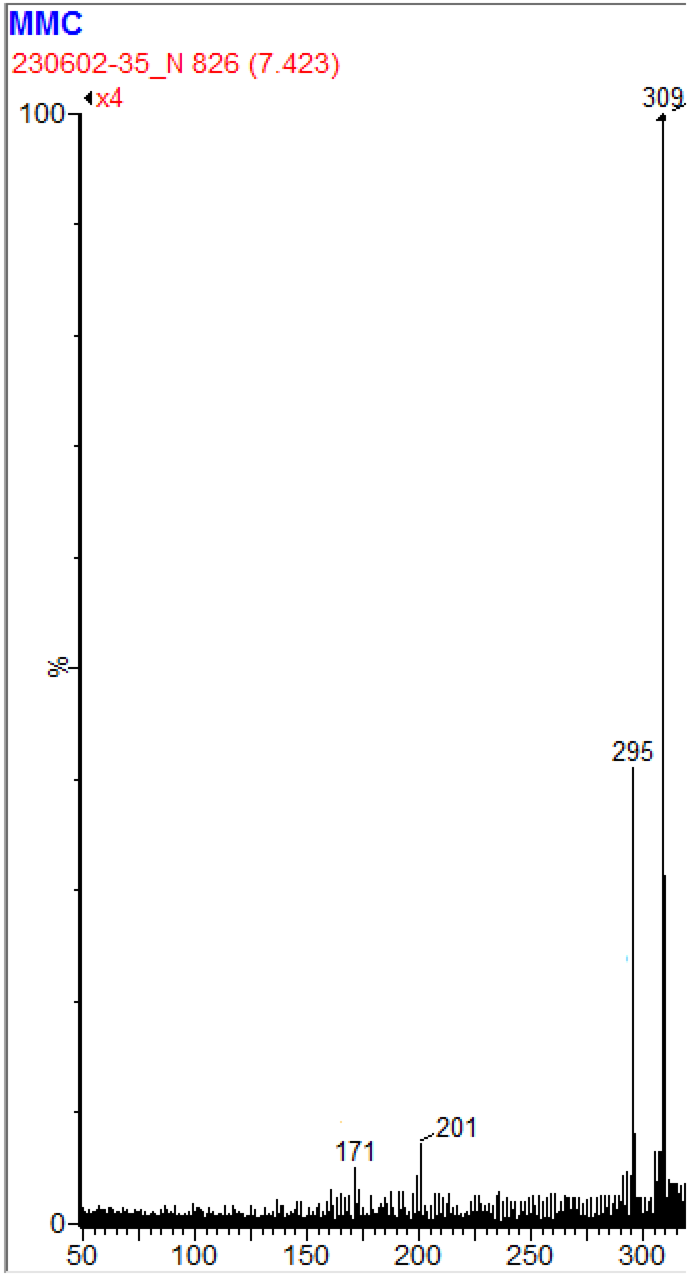

Dihydroxyoctadecenoic acid
[-]313.2406

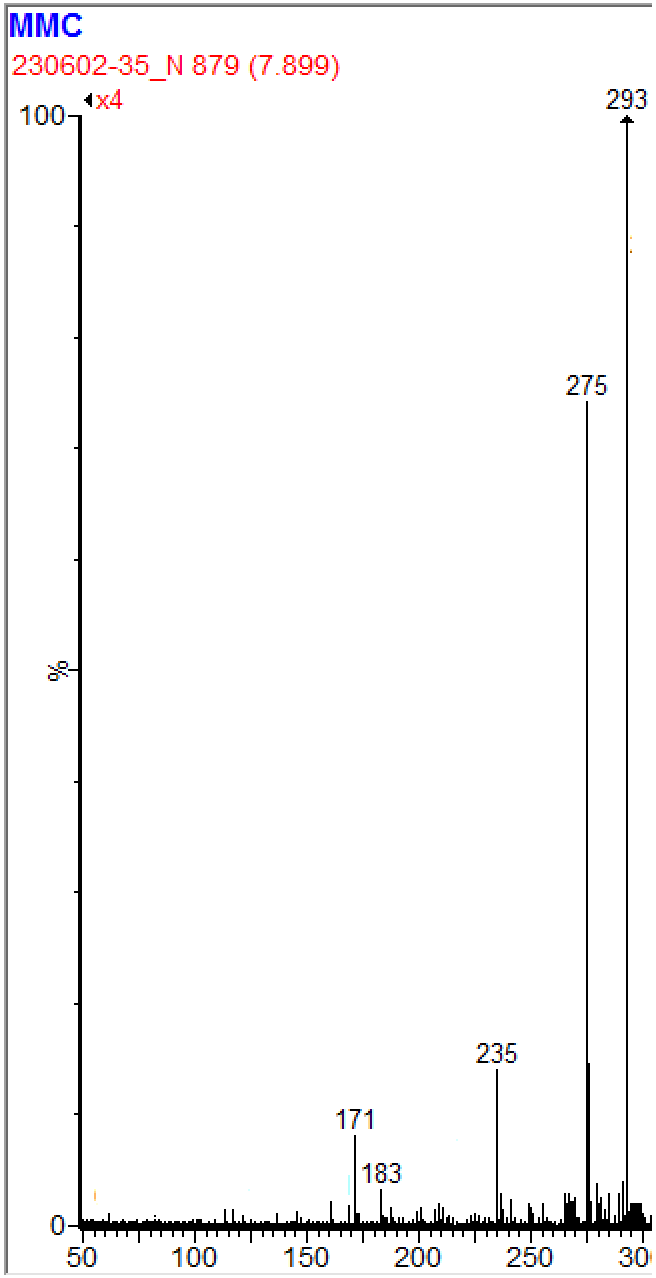

Hydroxyoctadecatrienoic acid
[-]293.2143

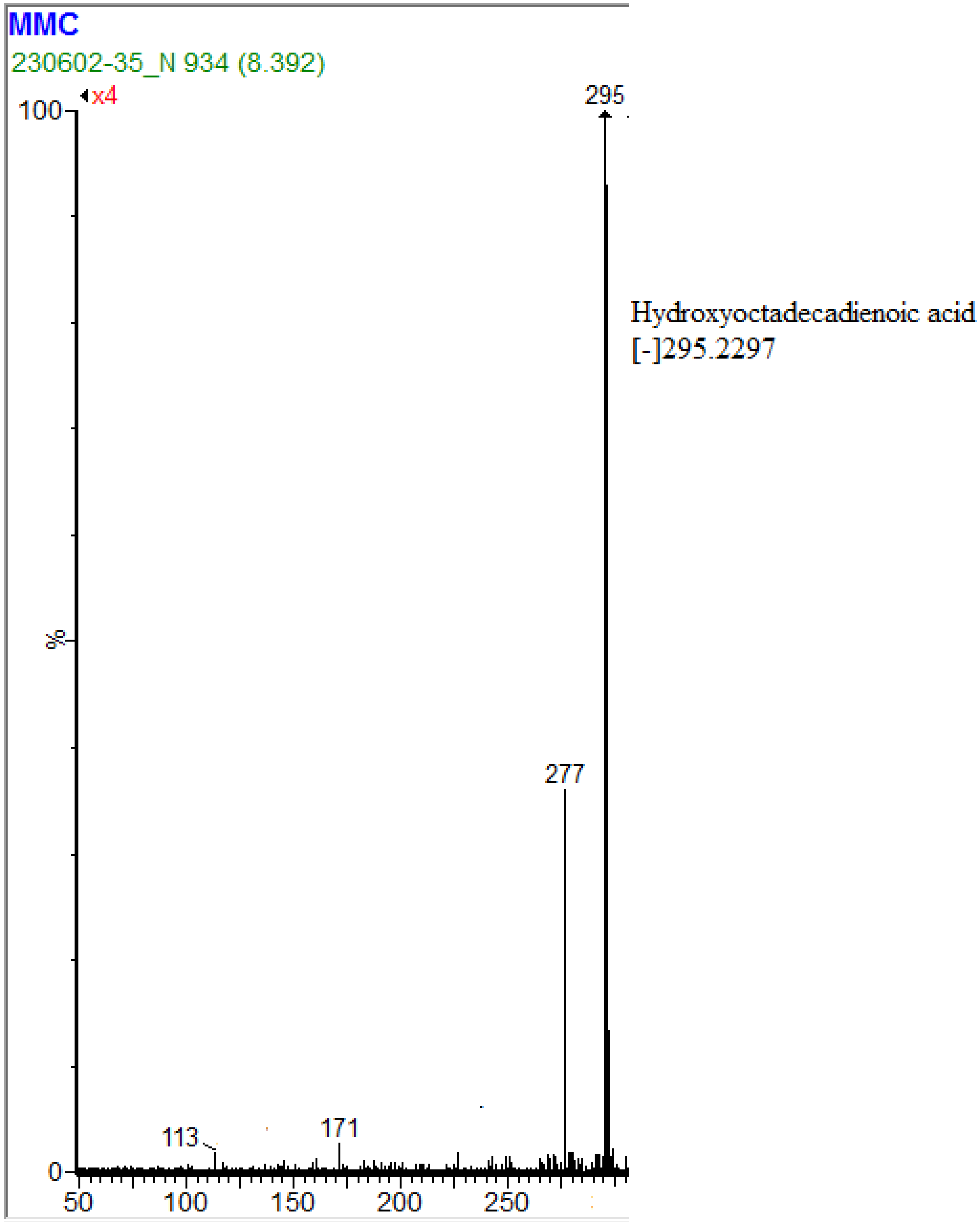

Hydroxyoctadecadienoic acid
[-]295.2297

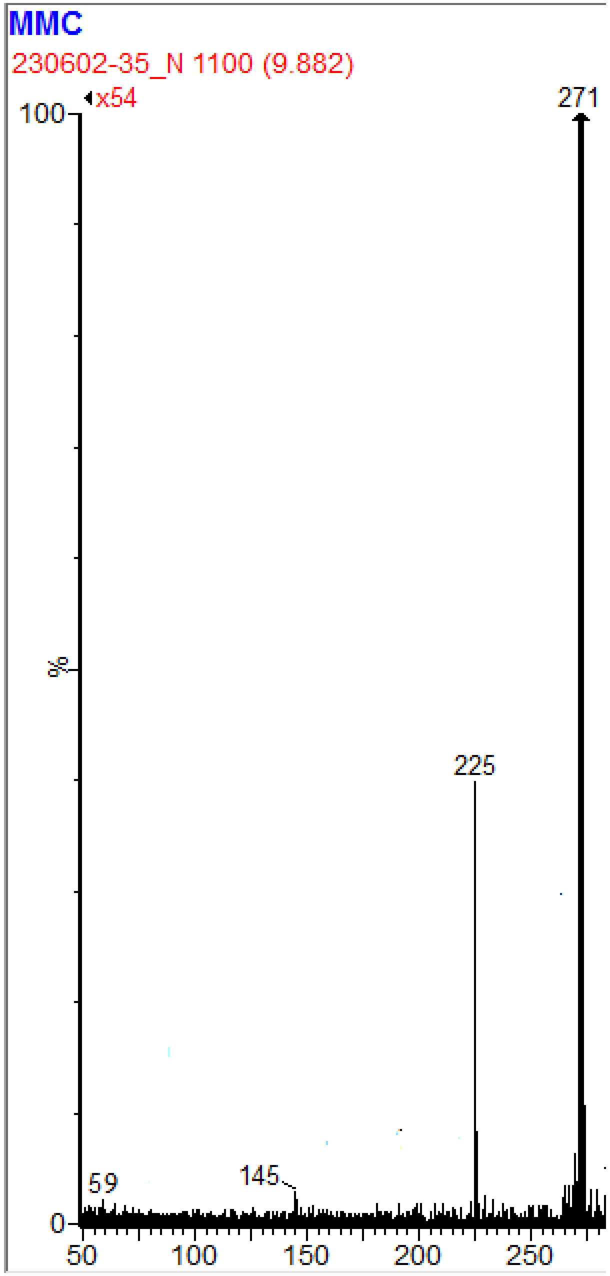

Hydroxyhexadecanoic acid (Juniperic acid)
[-]271.2281

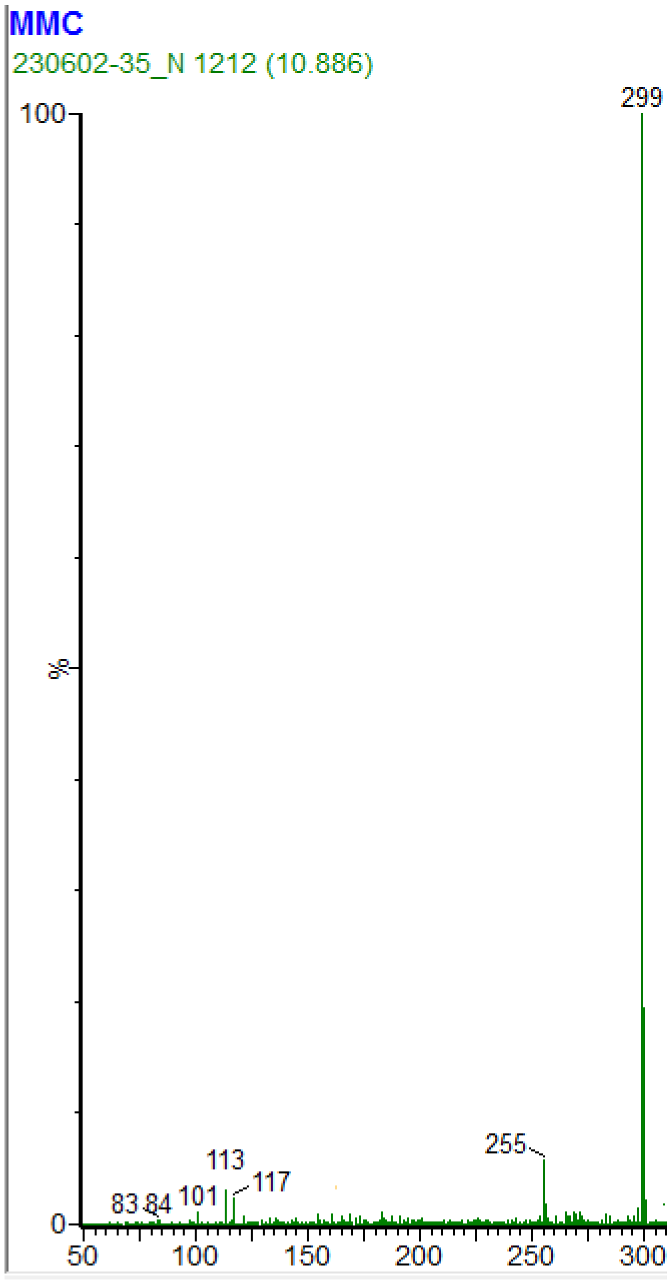

Hydroxy stearic acid
[-]299.2582

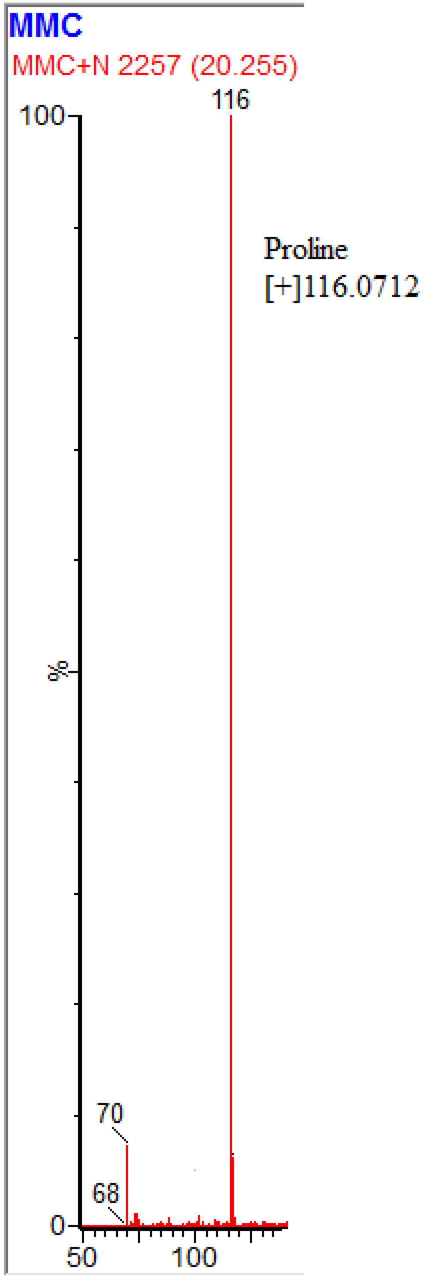

MMC
MMC+N 2257 (20.255)
100
116
Proline
[+]116.0712
%
70
68
0
50
100
